# *ADP1* Affects Plant Architecture by Regulating Local Auxin Biosynthesis

**DOI:** 10.1371/journal.pgen.1003954

**Published:** 2014-01-02

**Authors:** Ruixi Li, Jieru Li, Shibai Li, Genji Qin, Ondřej Novák, Aleš Pěnčík, Karin Ljung, Takashi Aoyama, Jingjing Liu, Angus Murphy, Hongya Gu, Tomohiko Tsuge, Li-Jia Qu

**Affiliations:** 1 State Key Laboratory of Protein and Plant Gene Research, Peking-Yale Joint Research Center for Plant Molecular Genetics and AgroBiotechnology, Peking-Tsinghua Center for Life Sciences, College of Life Sciences, Peking University, Beijing, People's Republic of China; 2 Department of Forest Genetics and Plant Physiology, Umeå Plant Science Centre, Swedish University of Agricultural Sciences, Umeå, Sweden; 3 Centre of the Region Haná for Biotechnological and Agricultural Research, Faculty of Science, Palacký University, Šlechtitelů 21, Olomouc, Czech Republic; 4 Institute for Chemical Research, Kyoto University, Gokasho Uji, Kyoto, Japan; 5 Department of Horticulture and Landscape Architecture, Purdue University, West Lafayette, Indiana, United States of America; 6 National Plant Gene Research Center (Beijing), Beijing, People's Republic of China; The University of North Carolina at Chapel Hill, United States of America

## Abstract

Plant architecture is one of the key factors that affect plant survival and productivity. Plant body structure is established through the iterative initiation and outgrowth of lateral organs, which are derived from the shoot apical meristem and root apical meristem, after embryogenesis. Here we report that ADP1, a putative MATE (multidrug and toxic compound extrusion) transporter, plays an essential role in regulating lateral organ outgrowth, and thus in maintaining normal architecture of *Arabidopsis*. Elevated expression levels of *ADP1* resulted in accelerated plant growth rate, and increased the numbers of axillary branches and flowers. Our molecular and genetic evidence demonstrated that the phenotypes of plants over-expressing *ADP1* were caused by reduction of local auxin levels in the meristematic regions. We further discovered that this reduction was probably due to decreased levels of auxin biosynthesis in the local meristematic regions based on the measured reduction in IAA levels and the gene expression data. Simultaneous inactivation of *ADP1* and its three closest homologs led to growth retardation, relative reduction of lateral organ number and slightly elevated auxin level. Our results indicated that *ADP1*-mediated regulation of the local auxin level in meristematic regions is an essential determinant for plant architecture maintenance by restraining the outgrowth of lateral organs.

## Author Summary

Plant architecture is one of the key factors that affect plant survival and productivity. It is well established that the plant hormone auxin plays an essential role in organ initiation and pattern formation, thus affecting plant architecture. We found that a putative MATE (multidrug and toxic compound extrusion) transporter, ADP1, which was expressed in the meristematic regions, through regulating the level of auxin biosynthesis, controls lateral organ outgrowth so as to maintain normal architecture in *Arabidopsis*. The more *ADP1* was expressed, the less levels of local auxin were detected in the meristematic regions of the plant, resulting in increased growth rate and a greater number of axillary branches and flowers. The reduction of auxin levels is probably due to decreased level of auxin biosynthesis in the local meristematic regions. Down-regulated expression of *ADP1* and its three closely related genes caused plants to grow slower and to produce less lateral organs. Our results indicated that *ADP1*-mediated regulation of the local auxin levels in meristematic regions is an essential determinant for plant architecture by restraining the outgrowth of lateral organs.

## Introduction

Higher plants have a diverse range of body structures. Phyllotaxis of lateral organs, branching pattern, as well as size, shape and position of lateral organs all contribute to the overall architecture of a plant. Plant architecture is the most obvious morphology of mature plants and has long served as an important criterion for systematic and taxonomic classification of plant species [1], [2]. Plant architecture is largely determined by genetic programs and, to some extent, by environmental cues, such as light, humidity, temperature, nutrition, and plant density. Detailed studies have been focused on genetic factors that are crucial for maintenance of shoot apical meristem (SAM), initiation and outgrowth of axillary meristem (AM), proper growth rate for lateral organ development, and correct timing for reproduction and senescence [2]. Research on plant architecture has important agronomic implications because it has a direct impact on the suitability and productivity of a plant. One of the most successful modifications of plant architecture is the Green Revolution, which is based on the selection of wheat cultivars with shorter and sturdier stems, resulting in plants with enhanced yield *via* improved resistance to wind and rain [3]. Over the past several years, branching patterns have been intensively investigated in rice, since the formation of tillers and panicle branches will greatly affect the efficiency of light absorption, which will in turn influence the adaptation of plants to the environment [4]–[6]. Understanding the genetic and molecular mechanisms of the regulation of plant architecture would help us to modify agronomically useful traits and thus facilitate the breeding of high-yield crops.

The success of the Green Revolution mainly results from selection of plants with altered biosynthesis and/or signaling of plant hormones, among which auxin is a determinant for plant architecture. Auxin is a critical factor controlling a wide variety of developmental processes, including embryogenesis, maintenance of apical dominance, and formation of lateral organs [7], [8]. Active auxin, mainly indole-3-acetic acid (IAA), is reported to be synthesized *de novo* by tryptophan (Trp)-dependent and/or independent pathways in the shoot apex, young leaves, and root apex [7], [9]–[14]. After synthesis, auxin is transported by the polar transport machinery [7], so that an appropriate distribution of auxin is established to maintain normal plant architecture. Disruption of auxin gradient, either by changing auxin biosynthesis, transport, or signaling, will lead to alteration of organ growth patterns and changes of plant architecture. For example, over-expression of the auxin biosynthesis genes *YUCCA1* (*YUC1*) and *YUCCA6* (*YUC6*) led to auxin over-production, resulting in increased apical dominance [15], [16], whereas the quadruple knock-out mutant *yuc1,2,4,6* showed abnormal flower development and loss of apical dominance, *i.e.*, increased branching [17], [18]. The double mutant *pgp1-1 pgp19-1* (*mdr1-1*), which exhibited a 70%–80% reduction in polar auxin transport, displayed pleiotropic phenotypes such as curly leaves, dwarfism and decreased fertility [19], [20]. Moreover, the auxin response mutant *axr1-12* and the *tir1 afb1 afb2 afb3* quadruple receptor mutant produced highly branched inflorescences at maturity. The quadruple mutant occasionally had no roots or produced only a single cotyledon, leading to lethality at early stages [21]–[23]. Thus, maintenance of proper auxin response and/or homeostasis is critical for normal plant architecture.

In this paper, we identified a dominant *Arabidopsis* mutant with an abnormal architecture, which we named *adp1-*D (*altered development program 1*- Dominant). The architecture of *adp1-*D was greatly altered at maturity, with increased number of axillary branches, flowers, and lateral roots. The growth rate of the mutant was accelerated throughout its life cycle. We discovered that the mutant phenotypes were caused by over-expression of *ADP1* gene. *ADP1* encodes a protein with sequence similarity to the multidrug and toxic compound extrusion (MATE) transporter family, which is found in prokaryotes and eukaryotes. MATE transporters are reported to be involved in a variety of important biological processes, since they function in the exclusion of toxic organic cation and disease resistance and exhibit multi-substrate specificity [24], [25].

Here we provide molecular and genetic evidence to demonstrate that the phenotypes of *adp1*-D were caused by reduction of the local auxin levels in the meristematic regions. The reduction was probably due to decreased levels of auxin biosynthesis in the local meristematic regions. When expression levels of *ADP1* and its three closest homologs were down-regulated in *Arabidopsis*, the resulting quadruple mutant exhibited growth retardation and a slight reduction of lateral organ number. Our results indicated that *ADP1* and its homologous genes play important roles in maintaining normal plant architecture, possibly by regulating local auxin biosynthesis.

## Results

### The *adp1*-D Mutant Displayed Pleiotropic Phenotypes

**Figure 1:**
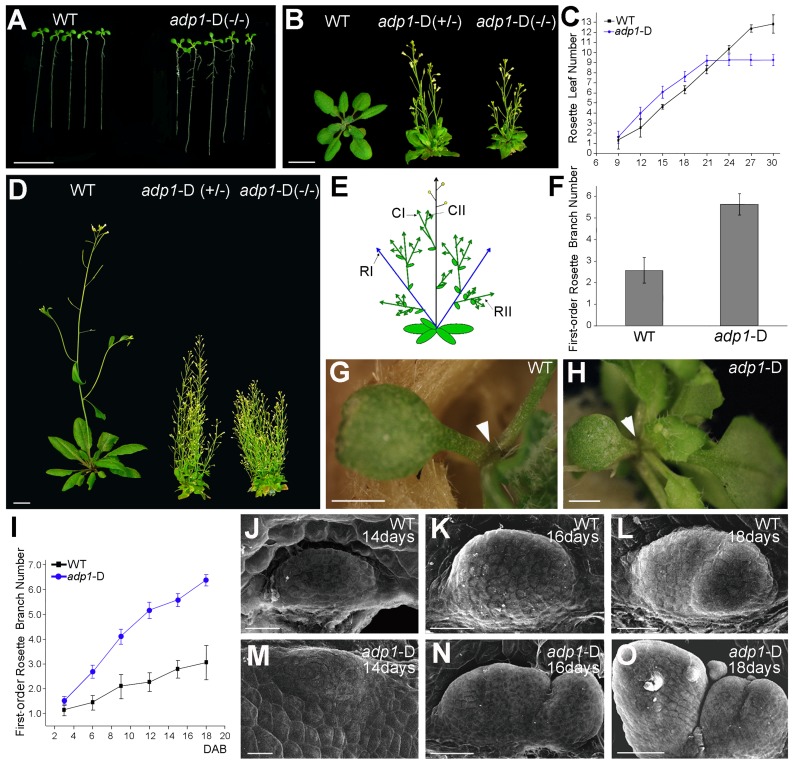
The *adp1*-D mutant displayed pleiotropic phenotypes. (A) Twelve-day-old seedlings and (B) 28-day-old plants grown under long-day conditions. The symbol +/− represents heterozygous mutants, while −/− represents homozygous mutants. (C) Emergence rate of rosette leaves in wild-type and *adp1*-D plants. At least 20 plants were measured for each genotype. (D) Six-week-old plants grown under long-day conditions. (E) Schematic diagram of *Arabidopsis* branching pattern. CI, first order cauline branches; CII, higher order cauline branches; RI, first order rosette branches; RII, higher order rosette branches. (F) First-order rosette branch number of two-month-old wild-type and *adp1*-D plants. At least 40 plants of each genotype were measured. (G) and (H) Detection of cotyledon petiole from 20-day-old plants of (G) the wild type and (H) the *adp1*-D mutant. Although no axillary bud was produced in the cotyledon axil in wild-type plants, about 30% of the *adp1*-D mutants produced an inflorescence in the cotyledon axil (indicated by arrowheads). (I) Generation rate of rosette branches in wild-type and *adp1*-D plants. At least 20 plants were measured for each genotype. Axillary buds longer than 2 mm were considered as axillary branches. (J) to (O) Scanning electron micrographs of developing axillary buds in the axils of the first pair of rosette leaves of wild-type (J–L), and *adp1*-D (M–O) plants. Plants were fixed after 14–18 days growth under long-day conditions. Development of the axillary buds can be divided into three stages. In stage 1, the axillary shoot bulged outwards, forming a semicircular zone. In stage 2, the outgrowth increased in size, forming an axillary leaf primordia. In stage 3, the axillary buds start to grow outwards. Under the growth conditions in this study, only one primordium was formed in the leaf petiole axil in wild-type plants, and most of the primordia stagnate in stage 2, whereas the axillary buds in *adp1*-D mutants developed faster (comparing K with N) and were at more advanced stages (comparing L with O). For (A) (B) and (D), bar = 1 cm; for (G) and (H), bar = 1 mm; for (J) to (O), bar = 40 µm.

We screened an *Arabidopsis* activation tagging mutant collection for mutants with altered plant architectures. The activation tagging mutant collection was generated using the activation tagging vector pSKI015 as described previously [26]. A dominant mutant with abnormal plant architecture, later designated *adp1*-D, was identified. The mutant displayed pleiotropic phenotypes, including accelerated growth rate of rosette leaves (Figure 1A, 1B and 1C), early flowering (Figure 1B and 1D), increased number of lateral roots (Figure S1A and Figure S1B). At maturity, the mutant had significantly more axillary branches (Figure 1D), including first-order rosette branches (RI, Figure 1E and 1F), higher-order rosette and cauline branches (RII and CII, Figure S1C). The lengths of first-order branches (RI and CI) were almost the same at different node positions (Figure S1D), suggesting the loss of apical dominance in the mutant. Occasionally, the axillary inflorescences were found in the axil of the cotyledons in the mutant (Figure 1H), which is unique because wild-type plants do not produce axillary inflorescences on the axil of the cotyledons (Figure 1G).

*adp1*-D mutant was almost sterile, producing very few seeds. Sterile phenotypes have been previously reported to be associated with induction of axillary branch outgrowth [27], [28]. In order to clarify whether the bushy phenotype of this mutant was a secondary effect of the sterile phenotype or not, we analyzed the kinetics of the initiation rate of the first-order rosette branches (RI). Our result showed that the difference between the mutant and wild type appeared as early as 3 days after reproductive transition, and the difference became larger with time (Figure 1I), until sterility appeared. However, exogenous application of GR24 [29], [30], a strigolactone-like inhibitor of shoot branching, revealed that the first-order rosette branches (RI) could be completely inhibited by GR24 application (Figure S2B to S2E), whereas higher-order cauline branches (CII) were not affected (Figure S2B to S2F), suggesting that the higher order branches were probably the secondary effect of sterility. Therefore in this study, we only focused on the first-order rosette branches.

To investigate the origin of the abnormal branches, we compared the early stage of axillary bud outgrowth in the first pair of leaves using scanning electron microscopy (SEM). In the wild type, although the axillary buds initiated from the epidermal cells in the semicircular zone, and then bulged outwards to form the meristems, in most cases, the axillary buds ceased development at this stage (Figure 1J to 1L). However, most of the axillary meristems of the mutant appeared to be larger than those of the wild type, and they continued to develop into inflorescences (Figure 1M to 1O). In many cases, the mutant had increased number of axillary meristems (Figure 1O). Since the developmental program of the mutant had been changed from the beginning to the end of the entire life cycle, and since the plant architecture had been greatly altered, we named the mutant as *adp1*-D (*altered development program 1*- Dominant).

### The *adp1*-D Mutant Is a Gain-of-Function Mutant

**Figure 2:**
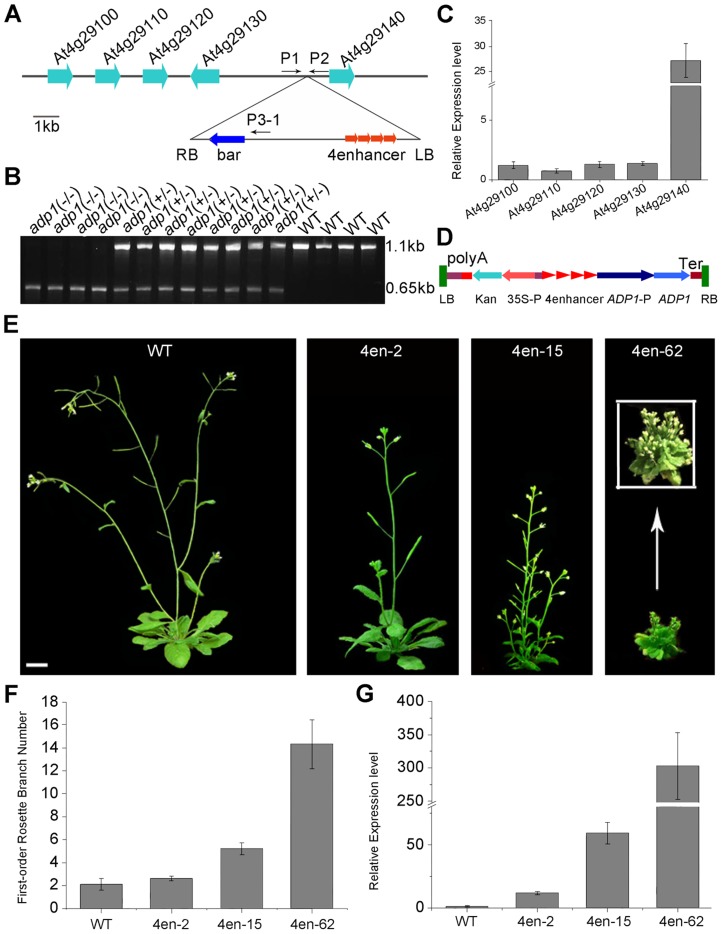
Characterization of the *ADP1* gene. (A) Schematic diagram of the genomic region flanking the T-DNA insertion site in *adp1*-D. The arrow direction represents the transcriptional orientation of the gene. The four red arrowheads represent the four 35S enhancers from pSKI015. LB, T-DNA left border; bar, Basta resistance gene; 4Enhancers, CaMV 35S enhancer tetrad; RB, T-DNA right border. (B) Linkage analysis of the T-DNA insertion and the bushy phenotypes. The primers P1 and P2 amplified an 1123-bp fragment from the wild type, and P1 and LBb1 amplified a 650-bp fragment from the homozygous *adp1*-D mutant. (C) Expression of genes flanking the insertion site in the wild type and the homozygous *adp1*-D mutant measured by quantitative RT-PCR with a *Tubulin* gene as an internal control. The expression levels of each gene in the wild type were set as 1.0. Error bars represent the SD of three biological replicates. (D) Schematic diagram of the construct for *At4g29140* over-expression in plants driven by four 35S enhancers. LB, T-DNA left border; polyA, CaMV 35S poly(A); Kan^R^, kanamycin resistance gene *NPT II*; 35S-P, CaMV 35S promoter; 4Enhancer, CaMV 35S enhancer tetrad; *ADP1-P*, promoter of *ADP1*; *ADP1*, open reading frame of *ADP1*; Ter, nopaline synthase terminator; RB, T-DNA right border. (E) Transgenic plants over-expressing *At4g29140* driven by four 35S enhancers showed the accelerated growth rate and bushy phenotypes. Bar = 1 cm. (F) First-order rosette branch number in transgenic plants. (G) Quantitative analysis of the *ADP1* expression level in transgenic plants. The expression level of the transgenic plants was in accordance with the severity of the phenotypes. Error bars represent three biological replicates.

To investigate the cause for the pleiotropic phenotypes in *adp1*-D, we performed thermal asymmetric interlaced PCR (TAIL-PCR), and identified a single T-DNA insertion in the intergenic region between *At4g29130* and *At4g29140* (Figure 2A). To examine whether the T-DNA insertion co-segregated with *adp1*-D phenotypes, we genotyped the T3 plants produced by heterozygous T2 mutants. Among 420 T3 plants, 102 were wild type without the T-DNA insertion, 106 were homozygous, and 212 were heterozygous with the T-DNA insertion (Figure 2B). All of the plants that were homozygous and heterozygous with the T-DNA insertion showed accelerated growth and increased number of lateral organs, whereas all of the plants without the T-DNA insertion appeared normal, suggesting that the pleiotropic phenotypes in *adp1*-D were caused by this single T-DNA insertion. To determine which gene was altered in its expression level, we examined the expression levels of all the genes within 10 kb upstream and downstream of the insertion site by quantitative RT-PCR. Only one gene, *At4g29140*, was over-expressed (by about 25-fold), while the expression of all the other genes remained mostly unchanged (Figure 2C), suggesting that over-expression of *At4g29140* could be responsible for the *adp1*-D phenotypes. To confirm this, we over-expressed *At4g29140* in wild-type *Arabidopsis* under its own promoter with four copies of the 35S enhancer (Figure 2D). The transgenic plants recapitulated all the phenotypes in *adp1*-D. About 10% of the transgenic plants showed more severe phenotypes of smaller plant size, highly compact leaves, and many more branches (Figure 2E and 2F). The expression level of *At4g29140* correlated with the severity of the phenotypes (Figure 2E to 2G). These data indicate that the pleiotropic phenotypes of *adp1*-D were indeed caused by over-expression of *At4g29140*.

*ADP1* has no intron and encodes a protein of 532 amino acid residues (Supplemental Figure 3A), sharing sequence similarity with the *Arabidopsis* MATE proteins. These proteins are characterized by 11 to 13 transmembrane helixes and two typical MATE domains [31]. The *Arabidopsis* genome contains 58 putative MATE transporters, which are grouped into five groups [32]. ADP1 belongs to a clade, which has eight members sharing high sequence identity in the conserved MATE domain (Figure S3B and S3C).

### *ADP1* Is Expressed in the Meristematic Regions

**Figure 3:**
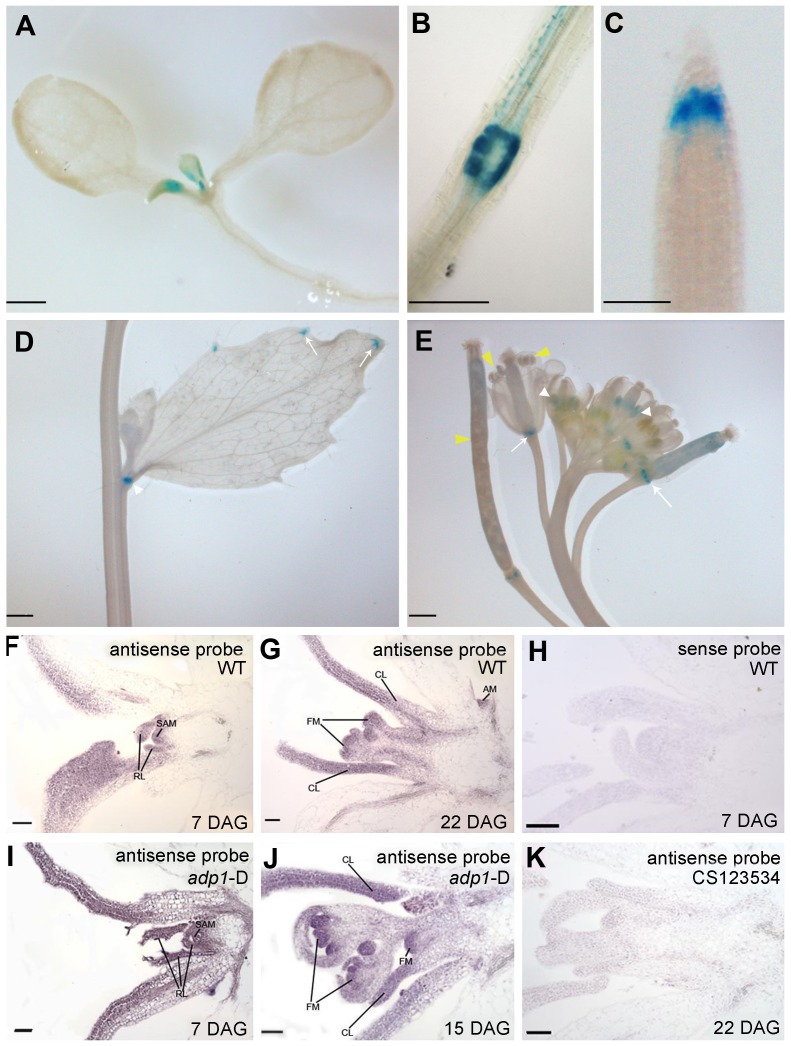
*ADP1* expression pattern. (A) to (E) *ADP1* expression pattern by Promoter:GUS staining. *ADP1* was expressed mainly in actively dividing tissues, such as leaf primordium and young leaf (A), the junction between lateral root and main root (B), root cap (C), the junction between cauline branch and the main inflorescence (D), the young stamen primordium and siliques (E, white and yellow arrow heads, respectively) and the junction between siliques and petiole (E, white arrows). (F) to (K) Wax-embedded sections of vegetative shoots and reproductive shoots from wild-type (F and G) and *adp1*-D (I and J) hybridized with the antisense probe. *ADP1* was expressed mainly in meristematic regions, such as the shoot apical meristem (SAM), axillary meristem (AM) in cauline leaf petiole and flower meristem (FM). RL, rosette leaf; CL, cauline leaf. (F) and (I), 8-day-old seedlings; (G), 22-day-old wild-type seedlings; (J), 15-day-old *adp1*-D seedlings. (H) Section from an 8-day-old wild-type seedling hybridized with the sense probe. (K) Section from a 22-day-old CS123534 seedling hybridized with the antisense probe. Bar = 20 µm.

To characterize the expression pattern of *ADP1*, a 2 kb promoter fragment upstream of *ADP1* start codon was fused with β-glucuronidase (GUS) reporter gene and transformed into wild-type *Arabidopsis*. GUS staining analysis of the homogenous transgenic lines showed that the promoter activity of *ADP1* was mainly detected in tissues where cells were actively dividing, such as leaf primordia and young leaves (Figure 3A), the junction between lateral root and the primary root (Figure 3B), root cap (Figure 3C), hydathodes (Figure 3D), the junction between secondary inflorescence and the main inflorescence (Figure 3D), young stamen and young siliques (Figure 3E).

Since the *adp1*-D phenotypes were apparently associated with shoot apical meristem (SAM) activity, we performed *in situ* hybridization for both wild-type and *adp1-*D seedlings. The transcript signals of *ADP1* were analyzed in shoot apical tissues, using a 300-bp *ADP1* cDNA fragment as the antisense probe. *ADP1* transcripts were detected primarily in the meristematic regions (*e.g.*, SAM), young leaves and flowers (Figure 3F and 3G). Comparison of *adp1*-D and the wild type showed that the overall distribution of mRNA was similar, but the signal in the *adp1-*D mutant was much stronger (Figure 3I and 3J compared with Figure 3F and 3G). No hybridization signals were detected for the control sense probe in wild type (Figure 3H), or the antisense probe in the *ADP1* loss-of-function mutant CS123534 (Figure 3K). Taken together these expression results correlated well with *adp1*-D phenotypes and indicated that *ADP1* transcript levels were up-regulated in *adp1-*D only within the regions where *ADP1* transcript was originally detected.

### ADP1 Is Localized in Endo-Membrane Structures

**Figure 4:**
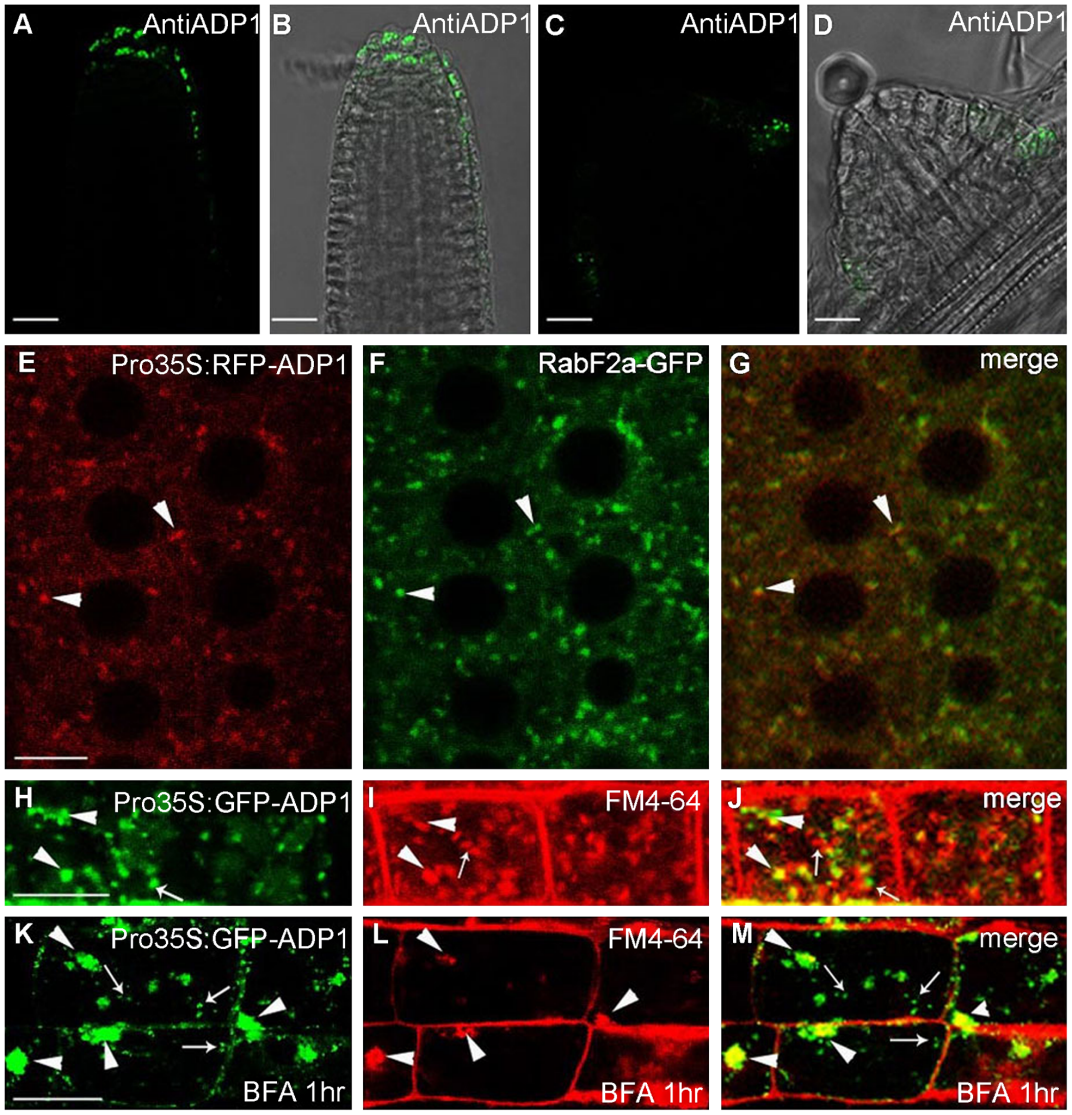
Subcellular localization of *ADP1*. (A) to (D) Subcellular localization of ADP1 in root cap (A and B) and the junction between lateral root and the main root (C and D). (E) to (G) RFP-*ADP1* was co-localized with *RabF2a-*GFP. Typical particles were indicated by arrowheads. (F) The merged image of the two fluorescence signals. (H) to (J) GFP-*ADP1* was partially co-localized with FM4-64 staining particles. The overlapped granules are indicated by arrowheads, and the non-overlapped granules are indicated by arrows. (K) to (M) GFP-*ADP1* was partially resistant to BFA treatment. The overlapped particles with BFA bodies of FM4-64 are indicated by arrowheads, and the resistant ADP1 granules are indicated by arrows. For (D) to (L), bar = 15 µm.

Antisera were raised against ADP1 and were used to immunolocalize ADP1 protein in the root apical meristem region. ADP1 was localized to small intracellular structures in the root cap and the junction between lateral root and primary root (Figure 4A to 4D). To further characterize these structures, transgenic lines were generated to express ADP1 in fusion with GFP or RFP on its N-terminus, under the CaMV 35S promoter. Approximately 30% of the transgenic lines with these tags recapitulated the mutant phenotypes to varying extents, suggesting that the fusion proteins were functional (Figure S4A to S4C). We found that, in the transgenic lines with recapitulated mutant phenotypes, the fluorescent signals of the fusion proteins were localized to punctuated particles with different sizes and shapes, which were distributed ubiquitously in the cells (Figure 4E), suggesting that ADP1 fusion proteins are retained in the endo-membrane organelles. To investigate the nature of these small particles, we crossed the endo-membrane marker lines with these transgenic lines, and found that ADP1 could co-localize with the endosome marker *RabF2a*-GFP [33] (Figure 4E to 4G) but not with TGN or ER markers (Figure S4D to S4J). Next, we used the fluorescent dye FM4-64 [34] to trace the endocytic dynamics of the fusion protein. After a half-hour treatment with the sterol dye FM4-64, the green signals (GFP tagged fusion protein) were partially co-localized with FM4-64 particles (Figure 4H to 4J). Next we treated the roots for one hour with brefeldin A (BFA), a fungal toxin that targets a subclass of ARF GEFs and is often used as an inhibitor of vesicle transport [35]. As a result, we found that some of the ADP1-GFP granules aggregated into larger bodies which co-localized with FM4-64, while the rest remained scattering in the cytoplasm (Figure 4K to 4M). Taken together, these results indicate that ADP1 resides in endo-membrane vesicles.

### Auxin Signals Were Decreased in Meristematic Regions in *adp1-*D

**Figure 5:**
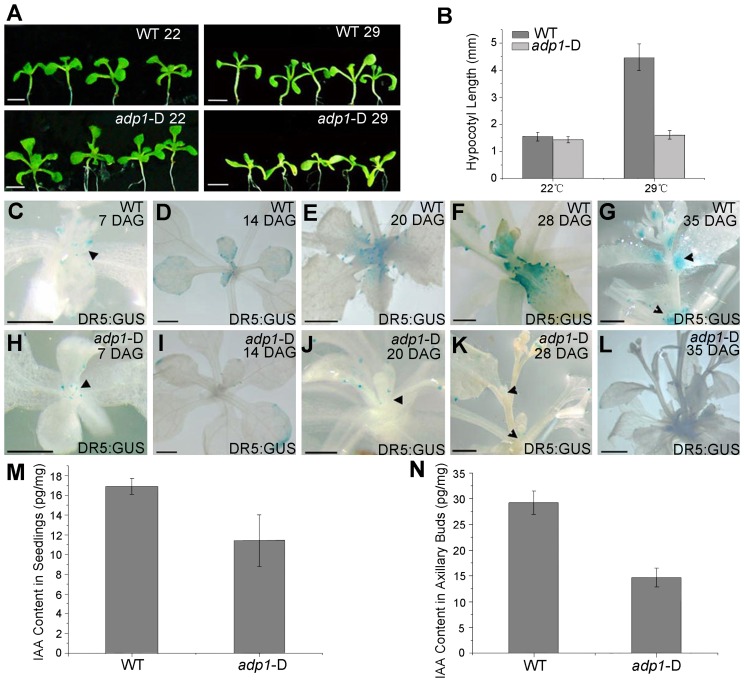
Evidence of reduced auxin levels in *adp1*-D mutants. (A) Morphology of wild type (WT) and *adp1*-D seedlings after growth for 9 days at 22°C (left) and 29°C (right) under long-day conditions. The white vertical line indicates the average hypocotyl length of wild-type and *adp1*-D seedlings. Bar = 5 mm. (B) Hypocotyl length of wild-type and *adp1*-D seedlings grown at 22°C and 29°C. At least 30 seedlings of each genotype were measured. The error bars represent the SD. (C) to (L) Detection of DR5:GUS signal in wild-type (C–G) and *adp1*-D (H–L) plants at different developmental stages. DR5:GUS stained mainly in the shoot apical meristem, young leaves and axillary buds in the wild-type plants (indicated by black arrowheads), whereas the signal in corresponding tissues of *adp1*-D was reduced dramatically. Bar = 0.5 mm. (M) and (N) Free IAA content in young seedlings (M) and axillary buds (N). 200 mg of corresponding tissues were dissected for the measurements and each experiment had three biological replications.

The phenotypes of *adp1*-D (*i.e.*, accelerated growth rate, highly branched shoots and increased number of lateral organs) resemble those mutants of auxin synthesis, transport, and response. To test whether auxin pathways are defective in *adp1*-D, we first examined hypocotyl length in a temperature shift experiment. The rationale was that decreased level of auxin, either by alteration of auxin biosynthesis, transport and/or signaling, would prevent hypocotyls from elongating under high temperature condition [36]. As shown in Figure 5A and 5B, after shifting the plants from 22°C to 29°C, the hypocotyl length of the wild-type seedlings increased by three to four folds, while that of *adp1*-D remained unchanged, suggesting that auxin synthesis, transport, or signaling was affected in the mutant. We then crossed *adp1*-D to DR5:GUS auxin-responsive reporter lines [37] and observed the GUS signal in different tissues of the F3 homozygous plants. In the wild type, DR5:GUS signals were detected mainly in the actively growing regions, such as the SAM, leaf tips, petiole bases, emerging axillary buds and flower primordia (Figure 5C to 5G). However, in *adp1-*D, DR5:GUS signals were substantially decreased in almost all the meristematic tissues (Figure 5H to 5L).

The decreased DR5:GUS signals indicated either decreased cellular auxin levels or altered auxin signaling. Root growth and the expression levels of two auxin-responsive genes (*IAA1* and *IAA5*) were examined in *adp1*-D after auxin treatment; an *adp1-*D *axr1-12* double mutant was also generated to test the genetic interactions of *ADP1* with auxin signaling mechanisms [21], [22], [38]. The results of these experiments indicated that *ADP1* does not interact directly with auxin signaling mechanisms (Figure S5).

The reduced auxin levels in *adp1-*D were further confirmed by direct quantitation of free IAA levels. Free IAA levels were measured in seedling shoot apices and axillary buds after bolting with quantification methods described previously [39]. The results showed that the free IAA content was indeed reduced in active dividing tissues in *adp1-*D (Figure 5M and 5N). Furthermore, sensitive mass spectrometry-based method of auxin metabolome profiling was conducted [40] to show that levels of several precursors [indole-3-acetonitrile (IAN), indole-3-acetamide (IAM), indole-3-pyruvic acid (IPyA) and indole-3-acetaldehyde (IAAld)] of auxin biosynthesis in *adp1-*D were decreased (Table S1). The same trend was also found in *adp1-*D, in terms of the free IAA level. These results are consistent with the hypothesis that enhanced outgrowth of axillary meristem might be caused by reduction of local auxin levels in *adp1*-D.

Although the decreased DR5:GUS signals might be caused by down-regulated auxin biosynthesis, the bushy phenotype of *adp1-*D is somewhat reminiscent of that of the *pgp1 pgp19* (*abcb1 abcb19*) mutants with impaired polar auxin transport. Furthermore, in *abcb19* mutant, that is defective in rootward polar auxin transport, levels of IAA at the shoot apex were decreased while levels of oxIAA and oxIAA-Glc were highly increased, as a result of long-term IAA pooling in this region. Accordingly, the polar auxin transport capacity in inflorescences and seedlings of *adp1*-D and wild type [41] were examined, revealing no differences for ^3^H-IAA (Figure S6). Due to the fact that this type of transport assay might not reveal differences in the transport capacity in the shoot apical meristem region, microscale transport assays [42] were used to determine if auxin transport capacity out of the meristem/cotyledonary node is affected in *adp1-*D. No difference between wild type and the mutant was detected by this method (data not shown), indicating that auxin transport is not impaired in *adp1-*D.

**Figure 6:**
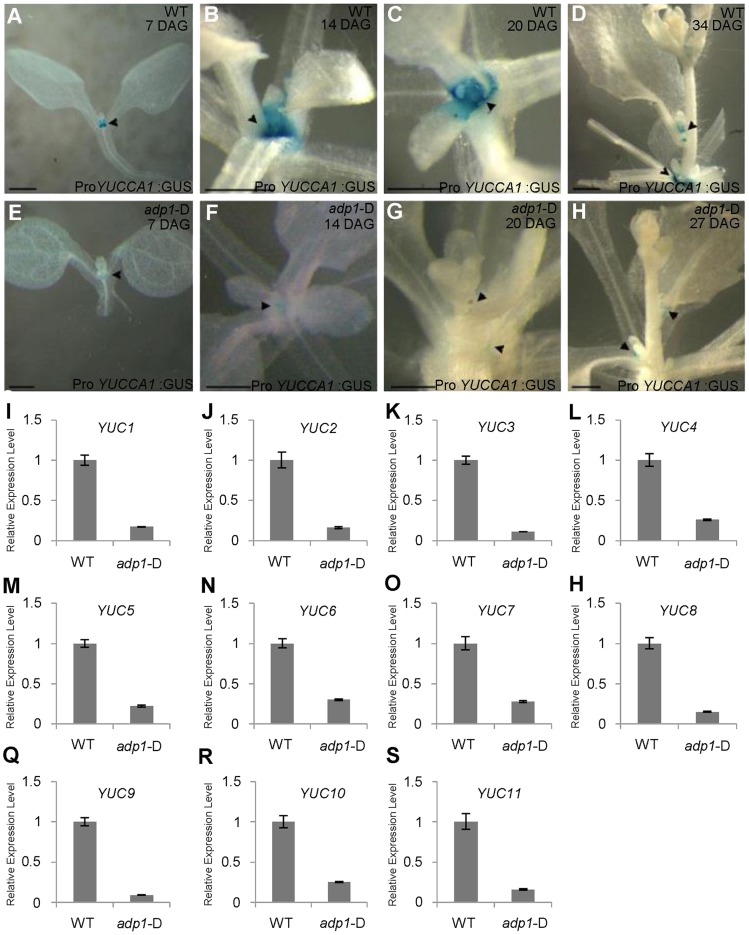
Auxin Biosynthesis was reduced in *adp1*-D mutants. (A) to (H) Detection of Pro*YUCCA1*:GUS signal in wild-type (A–D) and *adp1*-D (E–H) plants at different developmental stages. The Pro*YUCCA1*:GUS staining pattern was similar to that of DR5:GUS, i.e., mostly in young meristematic regions, as indicated by black arrowheads. The signal in corresponding tissues of *adp1*-D (indicated by black arrowheads) was reduced markedly. Bar = 0.5 mm. (I) to (S) Relative expression levels of all the *YUCCA* genes in wild-type and *adp1-D*. Axillary buds of adult plant (3 days after bolting) were collected as samples, and *Elongation factor-1α* was used as internal control. The expression levels of each gene in the wild type were set as 1.0. Error bars represent three biological replicates.

Analyses of auxin biosynthetic mutants suggested a connection of the observed phenotypes to the YUCCA family which belongs to flavin monooxygenase enzyme proteins functioning in auxin biosynthesis [10], [13]–[15], [17]. YUCCA6 has been shown to be associated with an endomembrane compartment [16]. The *yuc1 yuc4* double mutants and *yuc1,2,4,6* quadruple mutants, which are defective in auxin biosynthesis [17], [18], also exhibited reduced DR5:GUS signal in the SAM and in leaf petioles where axillary meristems were initiated (Figure S7A to S7H). These higher order *yuc* mutants also exhibited enhanced shoot branching (Figure S7I). Next, *adp1-*D was crossed with Pro*YUCCA1*:GUS transgenic marker lines. The GUS signal was decreased in almost all the meristematic regions in *adp1*-D, compared to that in the wild type (Figure 6A to 6H). This result indicated that *ADP1* over-expression affected auxin biosynthesis through the down-regulation of *YUCCA* expression, at least by *YUCCA1*. Furthermore, we examined the expression levels of all the *YUCCA* gene family members by qRT-PCR in the axillary buds after bolting, where *ADP1* was highly expressed. Results showed that the expression levels of all the *YUCCA* genes were decreased by three to ten folds in the axillary buds of *adp1-*D (Figure 6I to 6S). These results confirmed that the auxin biosynthesis pathway might be down-regulated in *adp1-*D.

### Transgenic Restoration of Auxin Biosynthesis in *adp1*-D Restores Wild-Type Growth

**Figure 7:**
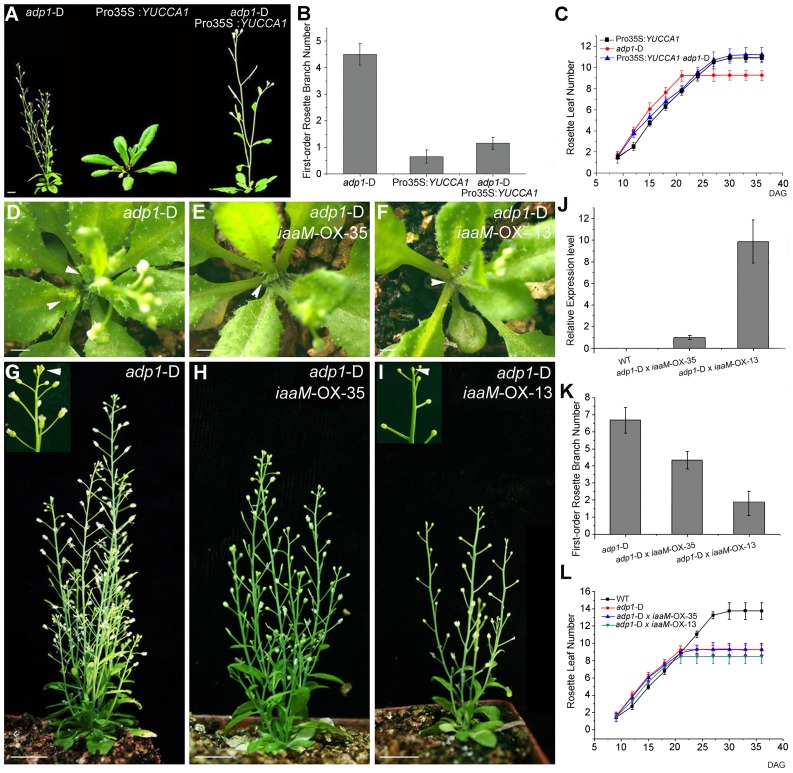
Correlation of auxin level with first-order rosette branch number. (A) Morphology of 35-day-old plants of *adp1*-D, Pro35S:*YUCCA1* and double mutants of *adp1*-D Pro35S:*YUCCA1*. Bar = 1 cm. (B) Quantitative analysis of first-order rosette branch from 2-month-old plants of *adp1*-D, Pro35S:*YUCCA1* and double mutants of *adp1*-D Pro35S:*YUCCA1*. Thirty plants of each genotype were measured. The error bars represent the SD. (C) Quantitative analysis of emergence rate of rosette leaves in *adp1*-D, Pro35S:*YUCCA1* and double mutants of *adp1*-D Pro35S:*YUCCA1*. Thirty plants of each genotype were measured. The error bars represent the SD. (D) to (I) Morphology of crossed lines between *adp1-*D and Pro*ADP1*:*iaaM* transgenic lines at 25 days after germination (D to F) and two months after germination (G to I). The axillary buds developed much slower in the crossed lines compared with *adp1-*D, indicated by arrowheads from (D) to (F). At maturity, the crossed lines with stronger *iaaM* expression level even showed terminated flowers, as indicated by the inserted frame in (I). (J) Expression quantity of *iaaM* in the wild type and the crossed lines shown in (D) to (I) analyzed by real-time-quantitative PCR (qPCR). (K) First-order rosette branch number of the wild type and the crossed lines shown in (D) to (I). (L) Emergence rate of rosette leaves in the wild type and the crossed lines shown in (D) to (I).

Crosses of *adp1*-D with a Pro35S:*YUCCA1* transgenic line that overproduces auxin [15] largely restored wild type growth (*i.e.*, both the rosette branch number and leaf initiation rate) in double homozygous F2 plants (Figure 7A to 7C). This result indicated that global increases of auxin levels through *YUCCA1* overexpression could rescue the pleiotropic phenotypes of the *adp1*-D mutant. In an effort to restrict the over-production of auxin to the expression domains of *ADP1*, the bacterial *indoleacetic acid-tryptophan monooxygenase* (*iaaM*) gene was expressed under the control of the *ADP1* promoter in wild type. iaaM catalyzes the conversion of Trp into indole-3-acetamide (the IAA biosynthetic precursor) [42], [43] and has been used to recapitulate Pro35S:*YUCCA1* phenotypes in *Arabidopsis* [15]. Domain-specific *iaaM* overexpression resulted in more epinastic leaves and aerial rosettes as well as reduced first-order rosette branch number (Figure S8A to S8I). qRT-PCR analysis showed that the severity of the phenotypes was correlated with *iaaM* expression levels (Figure S8H).

However, flower number and fertility were also reduced in Pro*ADP1:iaaM* lines, and, in some lines with severe phenotypes, the inflorescences were pin-formed or produced 3–4 undifferentiated flower meristems before termination (Figure S8B to S8D). These results suggest that overproduction of auxin in the *ADP1* expression domain can disrupt the sequential formation of auxin gradients which are required for normal floral development and phyllotactic growth [44]–[46]. Furthermore, these results indicate that auxin synthesis in the *ADP1* expression domains does not fully compensate for ADP1 transporter function in the endo-membrane system.

Based on phenotypes and relative gene expression levels, a stronger and a weaker Pro*ADP1*:*iaaM* transgenic lines were selected and crossed with *adp1*-D. Domain-specific expression of *iaaM* rescued the bushy phenotype in a manner corresponding to the expression level of *iaaM* in the parental lines (Figure 7D to 7K), but did not restore wild-type rosette leaf emergence rates (Figure 7L). These results suggest that increasing the auxin content in *ADP1* expression domains is sufficient to restore apical dominance, but is insufficient to overcome developmental defects resulting from reduced auxin production in *adp1-*D that produce accelerated first-order rosette branches.

### *ADP1* Accumulation Rescued the Pin-Formed Phenotypes of *pin1* and *pinoid*

The experiments described above indicated that ADP1 functions primarily in regulating auxin biosynthesis in the shoot apex and does not impact long-distance auxin transport capacity or auxin transport out of the SAM/cotyledonary node region. The *pinformed1* (*pin1*) mutant exhibits pin-formed inflorescences due to loss of the local auxin gradients that are mediated by the PIN1 auxin transporter at the shoot apex [47], [48]. Developmental and cell biology studies indicate that polar orientation of PIN1 in the SAM region follows and “canalizes” auxin gradients, which was generated by localized auxin biosynthesis and was associated with initiating floral primordia [49], [50]. Modeling analysis with PIN1-fluorescent fusion proteins and fluorescent auxin reporter indicates that these local gradients are formed successively to maintain phyllotactic growth [51], [52]. Since the PINOID (PID) kinase regulates PIN1 trafficking, polar localization of PIN1 is perturbed in *pid*, making *pid* form partial pin-formed inflorescences similar to *pin1* [53]–[55].

**Figure 8:**
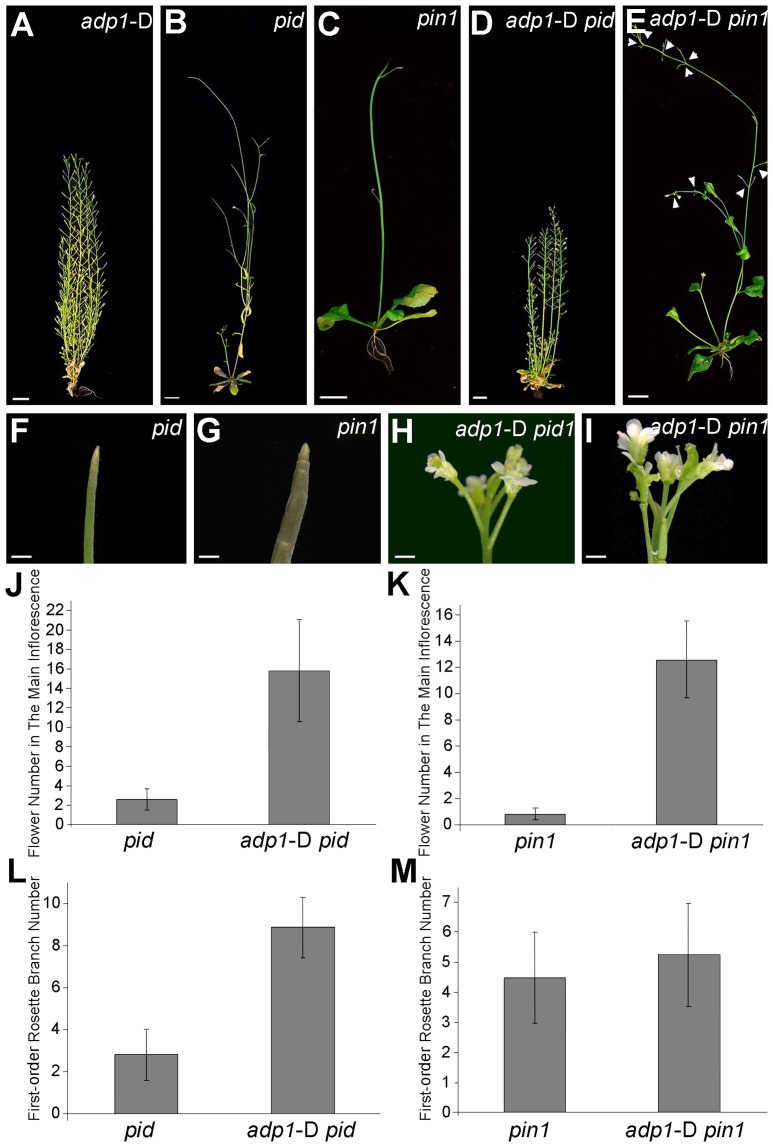
Overexpression of ADP1 partially recovered *pin1* and *pid* phenotypes. (A) to (E) Phenotypes of six-week-old *adp1*-D (A), *pid* (B), *pin1* (C) and double homozygous mutants *adp1*-D *pid* (D) and *adp1*-D *pin1* (E). Bar = 1 cm. (F) to (I) Close-up view of shoot apical tissue of *pid* (F), *pin1* (G), double homozygous mutants *adp1*-D *pid* (H) and *adp1*-D *pin1* (I). Hardly any flowers were formed in *pid* and *pin1*, but in the *adp1*-D *pid* and *adp1*-D *pin1* double mutants, much more flowers were produced on the inflorescence stem. Bar = 1 mm. (J) Flower number on the inflorescence stem of *pid* and *pid adp1*-D. At least 20 plants of each genotype were measured. The error bars represent the SD. (K) Flower number on the inflorescence stem of *pin1* and *pin1 adp1*-D. At least 20 plants of each genotype were measured. The error bars represent the SD. (L) First-order rosette branch number of *pid* and *pid adp1*-D. At least 20 plants of each genotype were measured. The error bars represent the SD. (M) First-order rosette branch number of *pin1* and *pin1 adp1*-D. At least 20 plants of each genotype were measured. The error bars represent the SD.

We hypothesize that the domain-specific decreases in auxin biosynthesis observed in *adp1-*D would weaken these auxin gradients, and thus would enhance the severity of the pin-formed phenotypes. Unexpectedly, the *adp1*-D *pid* double mutant displayed an overall appearance similar to *adp1*-D (Figure 8A, 8B and 8D), except that the mutant produced many flowers with fused petals (Figure 8F and 8H). Statistical analysis showed that the number of the flowers produced on the main inflorescence was at least three-fold more in the *adp1-D pid* double mutant than that in the *pid* mutant (Figure 8J), and the first-order rosette branch number was also increased by about two fold in the double mutant (Figure 8L). *ADP1* overexpression also largely rescued the *pin1* shoot phenotype, with greatly increased flower generation frequency in the inflorescence (Figure 8E, 8I and 8K). However, the first-order rosette branch number of the double mutants was not much increased (Figure 8M), probably due to the fact that *pin1* itself already has increased first-order rosette branch number, resulting from 30% decrease in auxin transport out of the shoot apex [20]. Taken together, these data indicate that increased *ADP1* activity at the shoot apex is sufficient to overcome a loss of PIN1-mediated canalization required for phyllotactic growth.

### Higher-Order Loss-of-function Mutants Exhibit Retarded Growth and Slightly Reduced Number of Lateral Organs

Seven homologous proteins were clustered with ADP1 in the same clade in the phylogenetic tree (Figure S3B and S3C), suggesting the possibility of functional redundancy. This is supported by an over-expression experiment in which each of these seven genes was driven under the CaMV 35S promoter to over-express each gene of interest in *Arabidopsis* plants. All of the transgenic plants recapitulated the *adp1*-D phenotypes in their T1 generation to different extents (Figure S9A to S9G).

**Figure 9:**
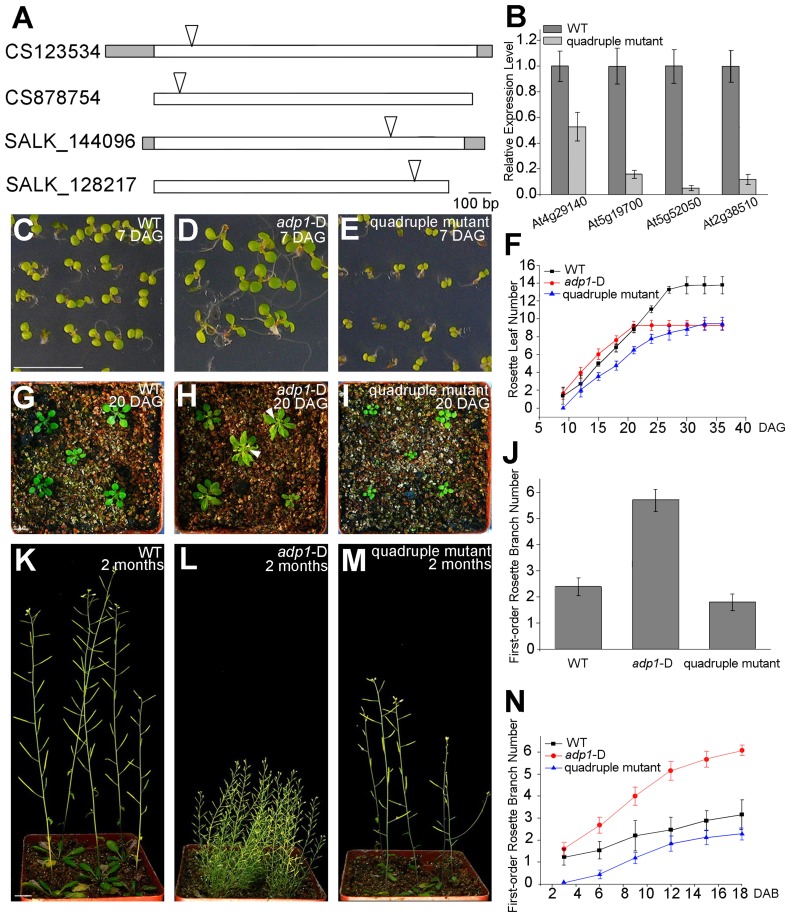
Growth retardance in the quadruple mutants. (A) T-DNA insertion sites in the CS123534, CS878754, SALK_144096 and SALK_128217 genes. Grey box, untranslated regions; white box, exon; arrowheads, T-DNA insertion sites. (B) Expression of *At4g29140*, *At5g19700*, *At5g52050* and *At2g38510* in wild-type seedlings and quadruple mutants as detected by real-time-qPCR. (C) to (E), (G) to (I) and (K) to (M) Morphology of wild-type seedlings, *adp1*-D and quadruple mutants at 7 days (C to E), 18 days (G to I) and 2 months (K to M) after germination, respectively. Bar = 1 cm. (F) Growth rate as measured by the record of rosette leaf emerging rate in wild-type plants, *adp1*-D and quadruple mutants. Forty plants of each genotype were measured. The error bars represent the SD. (J) First-order rosette branch number in mature plants of the wild type, *adp1*-D and quadruple mutants. Forty plants of each genotype were measured. The error bars represent the SD. (N) The generation rate of first-order rosette branch in the wild type, *adp1*-D and quadruple mutants. Forty plants of each genotype were measured. The error bars represent the SD.

To further elucidate the function of *ADP1* and its homologous genes, higher-order loss-of-function mutants were generated between the T-DNA insertion lines of *ADP1* and its closest homologs, *i.e.*, *At5g19700*, *At2g38510* and *At5g52050* (Figure S3B). The double mutants of each combination had no obvious phenotypes. However, some combinations of the triple mutants and the quadruple mutants exhibited developmental defects. In contrast to the gain-of-function mutant *adp1*-D, the quadruple mutants showed retarded growth from early developmental stages to maturation (Figure 9C to 9I and 9K to 9M). First-order rosette branch number was also slightly reduced in quadruple mutants compared to wild type (Figure 9J), and first-order rosette branches were generated much more slowly than the wild type (Figure 9N).

**Figure 10:**
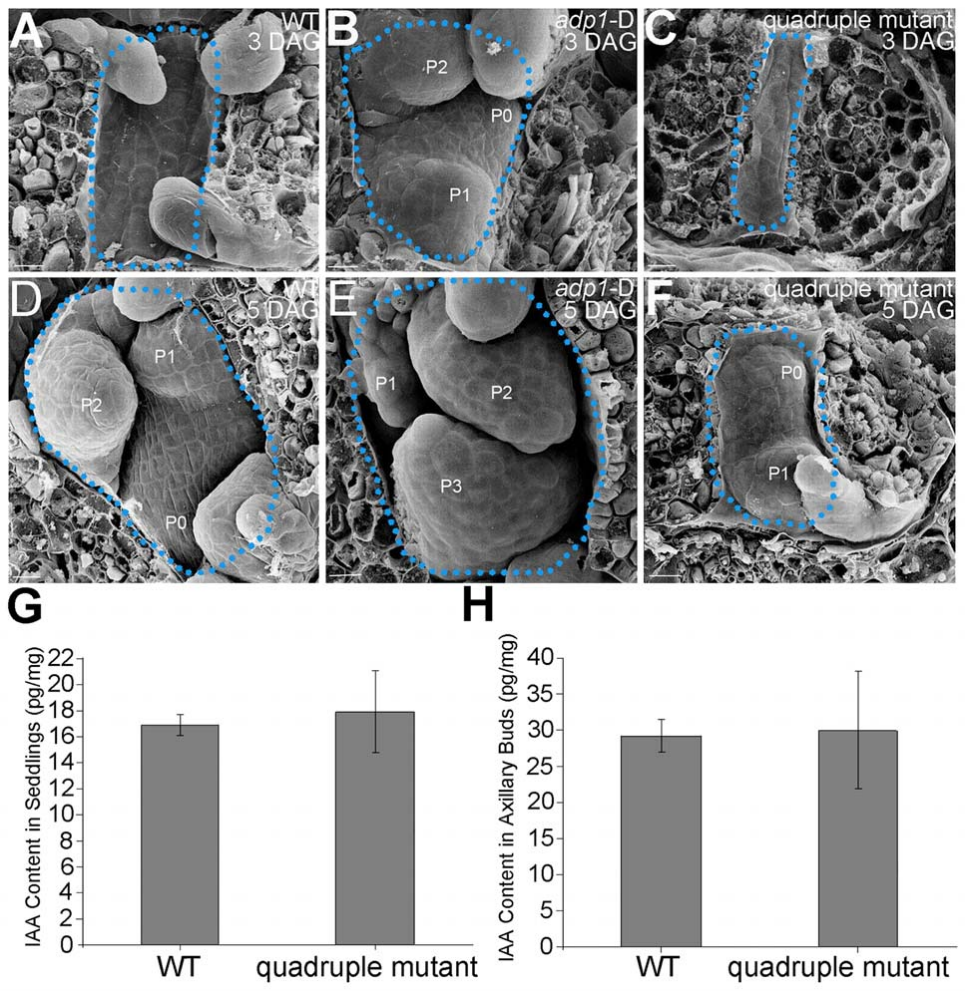
Morphology of Shoot apical meristems and detection of auxin level in *adp1*-D and quadruple mutants. (A) to (C) Scanning electronic micrographs of 3-day-old seedlings of (A) the wild type, (B) *adp1*-D, and (C) quadruple mutant. The area of apical tissues was enclosed by dashed lines. P0 to P3 indicate the leaf meristem by emergence order. Bar = 10 µm. (D) to (F) Scanning electronic micrographs of 5-day-old seedlings of (D) the wild type, (E) *adp1*-D and (F) quadruple mutant. Bar = 10 µm. (G) Measurement of IAA content in shoot apical tissues of seedlings from wild type and quadruple mutants. 200 mg of each genotype was dissected for the measurements and bars represent three biological replications. (H) Measurement of IAA content in axillary buds of wild type and quadruple mutants. 200 mg of each genotype was dissected for the measurements and bars represent three biological replications.

To clarify the origin of the aberrant growth pattern, we first examined the shoot apical regions of the wild type, *adp1*-D and the quadruple mutants by SEM. The results showed that the difference of the phenotypes between wild type, the quadruple mutants and *adp1-*D started as early as three days after germination (DAG). While *adp1-*D increased the size of the shoot apical region and produced more leaf primordia, the quadruple mutants showed much reduction of shoot apical size and retarded leaf initiation at 3 DAG (Figure 10A to 10C). This difference persisted from 3 DAG to 5 DAG (Figure 10D to 10F), suggesting that the developmental defects in the quadruple mutants and *adp1-*D reflect *ADP1* function rather than a concomitant developmental effect. Free IAA content in young seedlings and axillary buds of the quadruple mutant was measured by quantification methods described previously [39], showing that the free IAA levels of the quadruple mutants was not significantly different from that of wild type (Figure 10G and 10H). Same results (Table S1) were obtained by using the sensitive mass spectrometry-based method of auxin metabolome profiling [40], which might be due to additional redundancy of untested MATE transporters. However, we were able to detect slightly increased levels of several precursors (IAN, IAM and IPyA) of auxin biosynthesis (Table S1) by the mass spectrometry-based method. These results were consist with the phenotypes of the epinastic cotyledon and slightly increased hypocotyl length observed in the quadruple mutants (Figure S10), since the same phenotypes were also observed in Pro35S:*YUCCA1* plants which was believed to have increased auxin levels [15].

## Discussion

Branching pattern is one of the main factors contributing to plant architecture. In *Arabidopsis*, the number of the first-order and higher-order branches determines the light harvesting efficiency. While the first-order rosette branch number is controlled by strigolactones, the mechanism that regulates higher-order branches is largely unknown. Because many bushy mutants also exhibit reduced fertility, it is proposed that the increased number of higher order branches may be the consequence of sterility. The evidence presented here demonstrates that, in the bushy mutant *adp1-*D, increased first-order rosette branch number is a directly result of ADP1 overproduction.

Reduced auxin levels, transport, and signaling have long been associated with overproduction of rosette branches. Higher order *yucca* (auxin biosynthesis), *abcb/pgp* (auxin transport), and *tir1/afb* (auxin perception) mutants develop more rosette branches [17], [23], [56]. In the present study, *adp1-*D mutants produced an increased number of first-order rosette branches (Figure 1D and 1F) and Pro*ADP1*:*iaaM* plants showed a reduced number of first-order rosette branches (Figure S8I). Furthermore, lower levels of free IAA in *adp1-*D and the rescue of the *adp1-*D rosette branch phenotype by crosses *adp1-*D with Pro*ADP1*:*iaaM* transformants confirmed that reduction of local auxin levels in the active dividing meristems caused the bushy phenotype of *adp1-*D.

The growth retardation observed in the quadruple mutants was opposite to *adp1*-D phenotypes, suggesting that the genes in the same clade are functionally redundant in the regulation of lateral organ outgrowth in *Arabidopsis*. Although axillary shoot branching was not obviously changed in the quadruple mutants, the generation rate of the first-order rosette branches was much more slower in the mutants, compared to wild type, which is also opposite to that in *adp1-*D. In terms of free auxin levels, no significant difference was found in quadruple mutants, compared with wild-type (Figure 10G, 10H, and Table S1). This is probably due to gene redundancy, since the other four homologous genes of *ADP1* are still functioning in the quadruple mutants, which may be able to maintain a proper auxin homeostasis in the whole plants. However, levels of the several precursors (IAM, IAN, and IPyA) of auxin biosynthesis were indeed increased in the quadruple mutants (Table S1). Taken together with the excess-auxin phenotypes observed in quadruple mutants (slightly increased hypocotyls and epinastic cotyledons), the auxin biosynthesis in quadruple mutants might be slightly increased. The fact that both gain-of-function and loss-of-function mutants change the plant growth pattern indicates that temporally and spatially appropriate expression of these MATE genes are essential for maintaining plant architecture.

The partial rescue of *pid* and *pin1* pin-formed phenotypes by crosses with *adp1-*D is more difficult to rationalize. It is quite possible that over-expression of *ADP1* actually increases auxin levels within the *ADP1* expression domain. This increase is probably sufficient to overcome a loss of PIN canalization in some cell types, but leads to increased auxin catabolism in the majority of cells in the *ADP1* expression domain. Similar situation was found in the *abcb19/pgp19* auxin transport mutant in which decreased auxin levels and increased oxIAA-Hex levels were observed and attributed to an effect of auxin pooling near the SAM [41]. Alternatively, the decrease of IAA levels in *adp1-*D could alleviate repression of primordia induction by auxin, or, more likely, generate micro-gradients that stimulate development of new floral primordia.

ADP1 is a member of the large MATE family of transporters that have been implicated in mobilization of ions, toxins and secondary metabolites. As with many other transporter families, MATEs have expanded in plants and function in many aspects: the sequestration of a diverse range of secondary metabolites in vacuoles or their excretion out of the cells, and defense against herbivores and microbial pathogens [57]–[61]. One of the best characterized MATE transporters is TRANSPARENT TESTA12 (TT12), which has been shown to transport proanthocyanidin across the tonoplast [57], [61]. However, a number of MATE proteins appear to regulate the transport of organic acids. FRD3/AtDTX43 controls responses of iron deficiency in plant and is thought to mediate citrate secretion into the xylem and the rhizosphere [60]. The EDS5/AtDTX47 MATE protein functions in salicylic acid signaling for disease resistance [59]. ALF5/AtDTX19 was reportedly involved in the regulation of lateral root formation [58], suggesting a potential role in auxin signaling.

The results presented here indicate that ADP1 is localized to a post-Golgi endomembrane compartment and acts upstream of, or co-ordinately with, YUCCAs in auxin biosynthesis. YUCCA6 was localized to a similar endo-membrane compartment [16], suggesting that ADP1 may function in mobilization of IAA precursors to YUCCAs (for conversion to IAA), or, less likely, movement of IAA out of the endosomal compartments. The function of ADP1 may be homeostatic and involve reversible activity, because a prokaryotic MATE, NorM, which has been crystallized from *Vibrio cholera*, may exhibit conformational change on substrate binding [32]. Alternatively, ADP1 may simply prevent adsorption of hydrophobic indolic compounds into endosomal membranes or export IAA out of the endo-membrane vesicles. ADP1 may also be involved in auxin cellular homeostasis, which is maintained by PIN5 [62] and PILS auxin transporter [63]. Since so far no auxin exporter in ER has been reported except pollen specific PIN8 [64], it would be interesting to investigate in the future whether ADP1 could balance auxin homeostasis in ER. The lack of successful ADP1 protein expression in multiple heterologous expression systems (*e.g.*, different vectors in different *E. coli* strains, *S. cerevisiae*, *Pichea pastoris*, and SF9 insect cells) has prevented more detailed biochemical characterization.

There are multiple ADP1 homologues in rice, maize and sorghum, sharing 50% to 65% identity at the amino acid level. It is reasonable to speculate that over-expression of these *ADP1* genes could also change plant architecture in these crops. Crop plant architecture determines planting density in the field which, to a large extent, affects the light harvest, disease resistance, use of nutrients, and lodging [2]. Understanding the molecular mechanisms in the regulation of plant architecture will therefore provide a basis for modification of the plant architecture of crops, ultimately facilitating crop production.

## Methods

### Plant Materials and Growth Conditions

Seeds of *Arabidopsis thaliana*, ecotype Columbia, were surface sterilized with 15%? NaClO, stratified for 3 days at 4°C before incubation on Murashige and Skoog (MS) medium containing 1% sucrose at 22±2°C under long-day conditions (16 h light/8 h dark) for 1 week. Seeds of *adp1*-D mutants were sown on MS medium containing DL-phosphinothricin and drug-resistant seedlings were transferred to soil and grown under the same conditions.

For the temperature transferal experiment, plants were germinated and grown on MS medium for the wild type, and on MS medium containing DL-phosphinothricin for the *adp1*-D mutant, for 3 days under long-day conditions and then transferred to the same MS medium in a test chamber at 20°C and 29°C for another 7 days before measurement.

For assay of root elongation as an auxin sensitivity test, seedlings were grown on vertically placed MS medium for the wild type, and on MS medium containing DL-phosphinothricin for the *adp1*-D mutant, under long-day conditions for 4 days before transferal to medium containing 0, 20 nM, 40 nM, 60 nM, 80 nM and 100 nM of synthetic auxin 2,4-D. Root length was measured after incubation for 6 days.

For GR24 treatment, *adp1-*D were germinated and grown on MS medium containing DL-phosphinothricin for 7 days under long-day conditions and then transferred to MS medium containing 5 µM GR24 or 5 µM acetone for 40 days before phenotype analysis.

### Primers and PCR Conditions

Genomic DNA was extracted from homozygous and heterozygous *adp1*-D mutants and the flanking sequence of the T-DNA insertion was determined by thermal asymmetric interlaced PCR [65]. The specific degenerate primers in the T-DNA border and the random primers for three sequential PCRs were used as described previously [26]. Three primers (P1, P2 and P3-1) were designed for co-segregation analysis. P1 and P2 corresponded to the genomic sequence flanking the T-DNA insertion and P3-1 corresponded to the T-DNA vector sequence (Figure 2A). The primer sequences were as follows: P1 (5′-ATC CCA CTA AAG CAC TGT CA-3′); P2 (5′-TTT AAG CTA CTT ACC GTT GA-3′); and P3-1 (5′-TTG GTA ATT ACT CTTTCT TTT CCT CC-3′). For cloning of *ADP1*, the primer pair ADP1-F (5′-ATG TGT AACCCA TCA ACA ACA-3′) and ADP1-R (5′-TTA ATA AAG CAC CGT GAT GC-3′) were designed according to the cDNA sequence from the National Center for Biotechnology Information database (accession number NM_119058). Two primers, ADP1-RT-F (5′-CGA ACC GGA CTC TTC CTC GA-3′) and ADP1-RT-R (5′-GGT GAG CAC CGAAGG CTT GA-3′), were designed based on the coding region sequences to detect transgene transcripts in overexpression lines. The primers designed for amplification of the auxin-responsive genes *IAA1* and *IAA5* were IAA1-F (5′-GCG TCA GAA GCA ACAAGC G-3′); IAA1-R (5′-TCC TTT GTA GCC TTC TCT CTC GGA-3′); IAA5-F (5′-AGA TCT TGC TTC CGC TCT GCA A-3′) and IAA5-F (5′-CCC AAG GAA CATCTC CAG CAA GC-3′), respectively. The primers for detecting the transcription level of endogenous auxin transporters were as follows: PIN1-F (5′-TAC GGC GGC GGACTT CTA CC-3′); PIN1-R (5′-CGG CGA GGA AAC GGA GGT TC-3′); PIN2-F (5′-AATGGC CGT GAA CCC CTC CA-3′); PIN2-R (5′-TTG ACG TTC TCG GCG TCA CG-3′); PIN3-F (5′-CGG TAG CCT CGA GTG GAG CA-3′); PIN3-R (5′-CCG CCG GAC CGAAAT TGG AG-3′); PIN7-F (5′-TCT ACA CCG TCC TCA CGG CG-3′); PIN7-R(5′-AAG TTC GAA AGC CGG CCA CC-3′). The *TUB2* (β-tubulin) gene was used as an internal control in real-time PCR (qRT-PCR); the primers for *TUB2* used were those described previously (Qin et al., 2005). The qRT-PCR procedure comprised 40 cycles as described previously (Qin et al., 2003). PCR reactions were performed for 26–35 cycles (94°C for 30 s, 58°C for 30 s, and 72°C for 30 s to 1.5 min).

### Overexpression Constructs and *Arabidopsi*s Transformation

The *ADP1* cDNA was amplified from wild-type *Arabidopsis* by RT-PCR and cloned into the *Eco*R V site of the pBluescript SK+ vector (designated pBADP1). The presence of *ADP1* in the recombinant plasmids was confirmed by sequencing in both sense and antisense orientations. The *ADP1* promoter was amplified from genomic DNA using the primers 5′-GCT CAC AGG AGC CTT ACT TAT-3′ and 5′-GAC GGT GAT GAT GATGAT GGT-3′ and cloned into the *Eco*R V site of pBluescript SK+ (designated pBADP1P).

The CaMV 35S enhancer tetrad was amplified from pSKI015 as described previously [66] and was designated pA4Ehancer. The *iaaM* gene was amplified from the plasmid pBJ36-*iaaM* with the primers 5′-ATG TCA GCT TCA CCT CTC CT-3′ and 5′-TAA TTT CTA GTG CGG TAG TTA- 3′ and then cloned into the *Eco*RV site of pBluescript SK+ (designated pBiaaM). For the construction of the plant expression vector, the pQG110 vector [66], pJIM19 vector and pBI101.3 vector were used. 4Enhancer-ADP1 was constructed by ligation of four DNA fragments: the *Hind*III-*Xba*I fragment from pQG110, the *Hind*III-*Eco*RI enhancer tetrad fragment from pA4Ehancer, the *Eco*RI-*Kpn*I *ADP1* promoter fragment from pBADP1P, and the *Kpn*I-*Xba*I *ADP1* fragment from pBADP1. The Pro35S:*ADP1* construct was obtained by ligation of two DNA fragments: the *Kpn*I-*Sac*I fragment from pJIM19, and the *Kpn*I-*Sac*I *ADP1* fragment from pBADP1P. Pro:*ADP1-iaaM* was constructed by ligation of three DNA fragments: the *Xba*I-*Sa*cI fragment from pBI101, the *Xba*I-*Kpn*I *ADP1* promoter fragment from pBADP1P, and the *Kpn*I-*Sac*I *iaaM* fragment from pBiaaM. Wild-type plants were used for *Agrobacterium*-mediated transformation by the floral dip method. The seeds of transgenic wild-type plants were screened on MS medium containing 50 mg/mL kanamycin. The resistant seedlings were transferred to soil.

### *In situ* Hybridization

*In situ* hybridization was performed as described previously [67]. Antisense and sense probes were synthesized with digoxigenin-11-UTP (Roche Diagnostics) using T7 and T3 RNA polymerases, respectively. The primers used to amplify the DNA template for the probe synthesis were as follows: ADP1-INSITU-F (5′-ATG TGT AAC CCA TCA ACA ACA-3′) and ADP1-INSITU-R (5′-CGG TTA TGTTAG CAA AGG CAA T-3′).

### Histochemical Assays

GUS staining was performed in the following steps. Samples were first fixed in 90% acetone on ice for 20 min, then washed thoroughly three times with staining buffer (0.1 M Na_3_PO_4_, pH 7.0, 10 mM EDTA, 0.5 mM ferricyanide, 0.5 mM K ferrocyanide, and 0.1% Triton X-100) on ice, vacuum-infiltrated briefly, and incubated in staining buffer containing 50–100 mg X-gluc per 100 mL for 3–12 h.

### Tissue Sectioning and Microscopy

Tissue sectioning was performed as described previously [68]. Inflorescence stems were collected from the most basal 5 cm of stems of wild-type and *adp1-*D plants and fixed in FAA solution (50% ethanol, 5% acetic acid, and 3.7% formaldehyde). After dehydration with an ethanol gradient series, the samples were embedded in Historesin (Leica). Sectioning was performed using a Leica microtome and 7 µm sections were mounted on slides. The sections were stained with 0.25% (w/v) toluidine blue O (Sigma-Aldrich) and observed under an Olympus BX51 microscope as previously described [69]. Digital images were captured with a SPOT camera (Diagnostic Instruments) and processed using Adobe Photoshop.

### Marker Gene Analysis

*DR5*:*GUS*, Pro*YUC1*:*GUS*, Pro*PIN1*:*PIN1*:*GFP*, Pro*PIN2*:*PIN2*:*GFP*, Pro*PIN3*:*PIN3*:*GFP*, Pro*PIN7*:*PIN7*:*GFP*, Pro*PIN1*:*GUS* and Pro*PIN1*:*PIN1*:*GUS* marker lines were crossed to *adp1*-D and the homozygous lines in the T_3_ generation were used for analysis. In all analyses, the parental lines were used for comparison with those in the mutant background. Endo-membrane organelle localization and endocytotic dynamic analysis were performed as described previously [70].

### Generation of *adp1*-D Double Mutants

The double mutants *adp1*-D *axr1-12*, *adp1*-D *pin1* and *adp1*-D *pid* were generated by crossing heterozygous *adp1*-D with *axr1-12*, *pin1* or *pid*. The double mutants were identified from the F_2_ progeny grown in soil by comparison with the parental phenotypes and through PCR-based molecular analyses.

### Auxin Transport Assay

Auxin transport in the inflorescence stem was assayed as described previously [71]. Inflorescence stems of 6-week-old plants were cut into 2.5 cm segments, submerged with one end in a 1.5 ml microcentrifuge tube containing 30 µl MES buffer (5 mM MES, 1% [w/v] sucrose, pH5.5) with 100 nM _3_H-IAA in 1.45 µM total IAA at room temperature in the dark for 24 h. Basipetal or acropetal auxin transport was measured in accordance with the orientation of the inflorescence segments. After incubation, the segments were removed and the terminal 5 mm of the non-submerged ends were excised and placed into a scintillation vial containing 2.5 ml scintillation fluid for 18 h before counting with a liquid scintillation counter. Microscale auxin transport assays in seedlings were conducted as described previously [70].

### Measurement of IAA Content

For measurement of IAA content in seedlings, 7 days seedlings of *adp1-*D, wild type and quadruple mutants in long day conditions were sectioned with a sharp blade, and collected 200 mg tissues (including upper hypocotyls, shoot apical meristems and young leaves without cotyledons) for each genotype. For measurement of IAA content in axillary buds, 200 mg tissues of each genotype were collected three days after bolting, including 1 mm basal leave petiole and the attached newly produced axillary meristems. IAA content measurement was conducted as previously reported [39]. Mass spectrometry-based method of profiling the auxin metabolome [40] was conducted also, which requires much less amount of sample (approximately 20 mg). The samples were collected as the same way as for the above-mentioned method, except that only about 20 mg of each genotype was collected, and three biological replicates were conducted.

### Generation of Quadruple Mutants

The quadruple mutants were generated first by crossing CS123534 and CS878754, and crossing SALK_144096 and SALK_128217 to get the F_1_ heterozygous generation. Next, the two F_2_ homozygous mutants from the F_1_ selfed generation were crossed to obtain the F_3_ heterozygous mutants. Finally, the F_3_ selfed generation was screened by PCR to obtain the homozygous quadruple mutants in the F_4_ generation.

### Accession Data

Sequence data for *ADP1*, *YUCCA1*, *PIN1*, *PIN2*, *PIN3*, *PIN7*, *AXR1* and *PINOID* can be found in the GenBank/EMBL data libraries under accession numbers NM_119058, NM_119406.2, NM_106017.3, NM_125091.3, NM_105762.2, NM_102156.1, NM_001035893 and NM_129019.

## Supporting Information

Figure S1 Phenotypes of *adp1-*D. (A) Measurement of the lateral root number in 12-day-old seedlings of wild type and *adp1-*D plants. Thirty plants of each genotype were measured. The error bars represent the SD. (B) Measurement of lateral root number at different root positions in 12-day-old seedlings of wild type and *adp1-*D. Lateral roots extended to more basal positions in *adp1-*D, compared with those in wild type. Thirty plants of each genotype were measured. The error bars represent the SD. (C) Higher-order branch number of two-month-old wild type and *adp1-*D plants. Thirty plants of each genotype were measured. The error bars represent the SD. (D) First-order branch length at different node positions of wild type and *adp1-*D plants. Node position number increased with the distance to the shoot apical meristem. The node length decreased dramatically from top to bottom in wild type plants and almost no visible branch could be detected at the fifth node, but in the mutant *adp1-*D, the branch length were almost the same, and extended to much lower position. Thirty plants of each genotype were measured. The error bars represent the SD.
(TIF)

Figure S2 GR24 treatment of *adp1-*D. (A) to (D) Morphology of *adp1-*D grown on MS with 0.1% acetone (A) or 5 µM of GR24 (B to D) for 40 days. GR24 treatment resulted in almost completely inhibition of first-order rosette branches in *adp1-*D, indicated by white arrows from (B) to (D). However, GR24 had little effect on higher-order cauline branches, indicated by white arrowheads. (A) First-order rosette branch number of *adp1-*D grown on MS with 0.1% acetone or 5 µM of GR24. Forty plants were measured. Error bars represent SD. (B) Higher-order branch number of *adp1-*D grown on MS with 0.1% acetone or 5 µM of GR24. Forty plants were measured. Error bars represent SD.
(TIF)

Figure S3 *ADP1* belongs to the MATE transporter family. (A) Schematic diagram of the *ADP1* cDNA structure. Grey bars represent the untranslated regions (UTR), the white bar represents the coding sequence (CDS). (B) Sequence alignment of eight genes belonging to the same clade as *ADP1*. Double asterisks indicate high identity, and a single asterisk indicates moderate similarity. The blue line indicates the sequence of the MATE domain. (C) Phylogenic relationships among eight genes from the same clade as *ADP1*.
(TIF)

Figure S4 Co-localization of *ADP1* with marker lines. (A) to (C) Morphology of 30 days' wild type plants (A), transgenic plants of Pro35S:GFP-ADP1 (B) and Pro35S:RFP-ADP1(C). (D) to (F) The fluorescent signal of GFP-*ADP1* did not co-localize with ER-RFP, as indicated by arrowheads (GFP-*ADP1*) and arrows (ER-RFP). Bar = 20 µm. (G) to (I) The fluorescent signal of RFP-*ADP1* did not co-localize with TGN-GFP, as indicated by arrowheads (TGN-GFP) and arrows (RFP-*ADP1*). Bar = 20 µm.
(TIF)

Figure S5 Auxin signal transduction pathway in *adp1*-D. (A) Primary root length in wild-type and *adp1*-D seedlings after growth on medium containing different concentrations of 2,4-D for 6 days. At least 30 seedlings were measured for each genotype. The error bars represent the SD. (B) *IAA1* and *IAA5* expression in wild-type and *adp1*-D seedlings after 1 h treatment with 20 µM 2,4-D. (C) Phenotypes of six-week-old *adp1*-D, *axr1-12*, and *adp1*-D *axr1-12* mutants. Bar = 1 cm. (D) First-order rosette branch number in the wild type, *adp1*-D, *axr1-12*, and *adp1*-D *axr1-12*. (E) Higher-order branch number in the wild type, *adp1*-D, *axr1-12*, and *adp1*-D *axr1-12*. For (D) and (E), at least 30 seedlings were measured for each genotype. The error bars represent the SD.
(TIF)

Figure S6 Auxin flux in the main stem did not change in *adp1*-D. (A), (B), (C), and (D) Transverse sections of vascular tissue in the basal portion of the inflorescence stem of A) and B) wild-type and C) and D) *adp1*-D plants stained with toluidine blue. C, cortex; If, inter-fascicular fiber; Pc, (pro) cambium; Pi, pith; Ph, phloem; Vb, vascular bundle; Xy, xylem. Bars = 100 µm. (B) and (D) Higher-magnification images of the vascular tissue in B) the wild type and D) *adp1*-D. (E) Polar auxin transport in the inflorescence stem of wild-type and *adp1*-D homozygous seedlings. Fifteen seedlings of each genotype were assayed. Values shown are means ± SD. (F) and (G) Measurement of first-order rosette branch number (F) and higher-order branch number (G) of *adp1*-D plants after cultivation for six weeks on MS medium containing 0.1% DMSO, 0.5 µM, 1 µM, or 2 µM NPA. Twenty plants were measured in each treatment.
(TIF)

Figure S7 Reduction in DR5:GUS signal in *yuc1,2,4,6* quadruple mutants. (A) to (H) DR5:GUS signal at different developmental stages in the wild type (A, C, E, G) and *yuc1,2,4,6* mutants (B, D, F, H). The GUS signal (indicated by black arrowheads) was almost undetectable in meristematic regions in the quadruple mutants compared with that in the wild type. For (A) to (F), bar = 0.1 mm; (G) and (H), bar = 0.4 mm. (I) Phenotypes of 2-month-old wild type and *yuc1,2,4,6* plants. Bar = 1 cm.
(TIF)

Figure S8 Correlation of auxin level with lateral organ number. (A) to (G) Morphology of transgenic plants carrying the control vector (A) or Pro*ADP1*:*iaaM*. The epinastic leaf is indicated with a white arrow; aerial rosette leaves are indicated by white arrowheads; terminated shoot tips are indicated by yellow arrowheads; and sterile siliques are indicated by blue arrowheads. Bar = 1 cm. (H) Expression quantity of *iaaM* in the transgenic lines shown in (A) to (G) analyzed by real-time-qPCR. (I) First-order rosette branch number of transgenic lines shown in (A) to (G). (J) Flower number on the main of transgenic lines shown in (A) to (G).
(TIF)

Figure S9 Recapitulation of *adp1*-D phenotypes by over-expression of genes in the same clade. (A) to (G) Phenotypes of transgenic plants over-expressing genes belonging to the same clade as *ADP1* under the CaMV 35S promoter and the expression quantity in the corresponding lines (see legend below). All transgenic plants recapitulated the bushy and accelerated growth rate phenotypes of *adp1*-D to different extents. The expression level was in accordance with the severity of the phenotype. Phenotypes and expression level of transgenic plants over-expressing A) Pro35S:5g19700, B) Pro35S:4g23030, C) Pro35S:2g38510, D) Pro35S:5g52050, E) Pro35S:5g49130, F) Pro35S:1g71870 and G) Pro35S:1g58340. Bar = 1 cm.
(TIF)

Figure S10 The phenotypes of quadruple mutant. The quadruple mutant exhibited epinastic cotyledon (A) and increased hypocotyl length (B). Black arrows indicate epinastic cotyledons. The error bars represent the SD. Bar = 1 mm.
(TIF)

Table S1 IAA precursors and IAA levels (pg (ng^Δ^)/mg FW).
(DOC)
