# Supplementary figures and images for: ADP1 Affects Plant Architecture by Regulating Local Auxin Biosynthesis
Source: PLoS Genet. 2014 Jan 2;10(1):e1003954. doi: 10.1371/journal.pgen.1003954 (PMC3879159; doi:10.1371/journal.pgen.1003954)

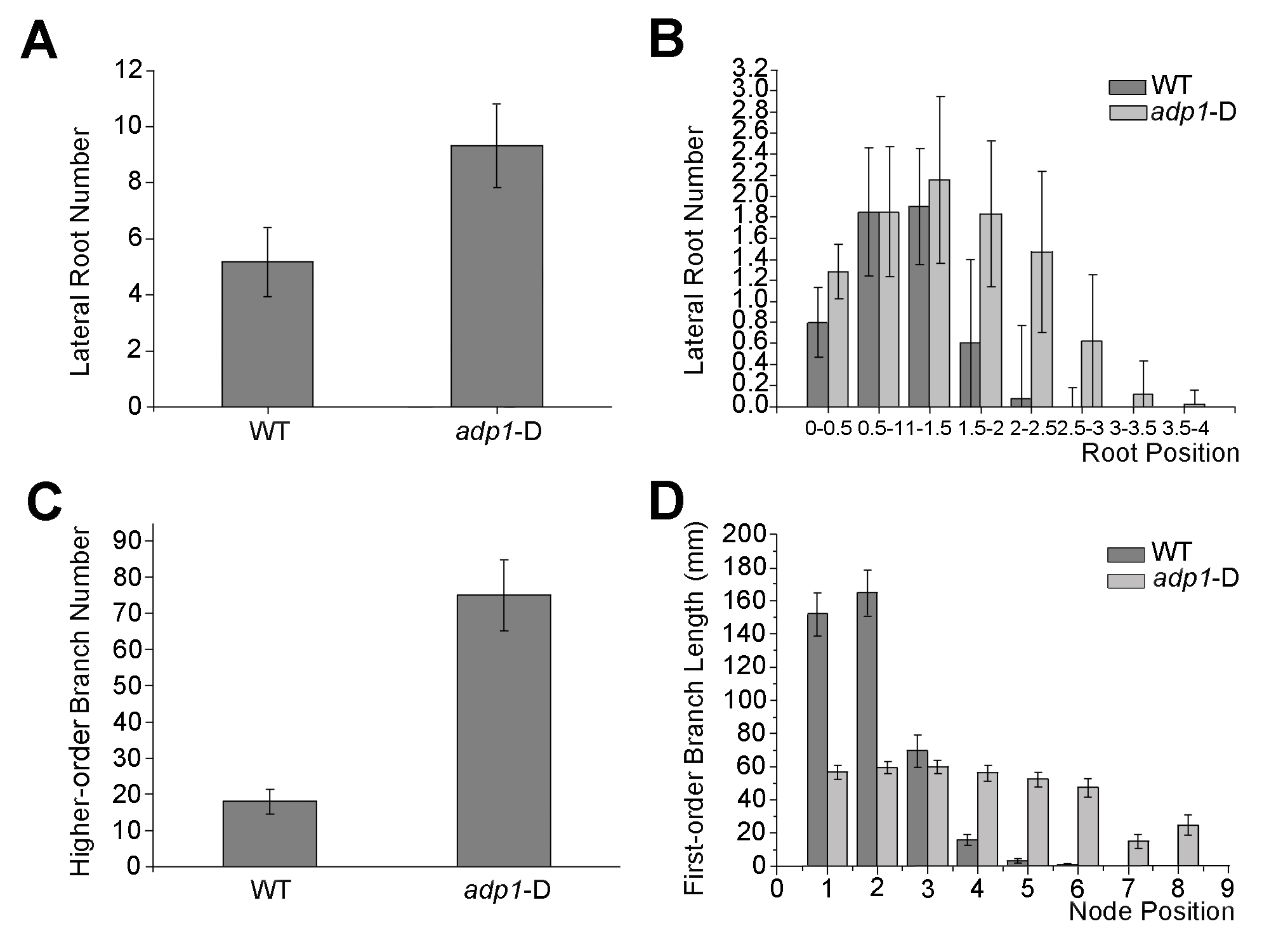

Supplement: Figure S1 — Phenotypes of adp1-D. (A) Measurement of the lateral root number in 12-day-old seedlings of wild type and adp1-D plants. Thirty plants of each genotype were measured. The error bars represent the SD. (B) Measurement of lateral root number at different root positions in 12-day-old seedlings of wild type and adp1-D. Lateral roots extended to more basal positions in adp1-D, compared with those in wild type. Thirty plants of each genotype were measured. The error bars represent the SD. (C) Higher-order branch number of two-month-old wild type and adp1-D plants. Thirty plants of each genotype were measured. The error bars represent the SD. (D) First-order branch length at different node positions of wild type and adp1-D plants. Node position number increased with the distance to the shoot apical meristem. The node length decreased dramatically from top to bottom in wild type plants and almost no visible branch could be detected at the fifth node, but in the mutant adp1-D, the branch length were almost the same, and extended to much lower position. Thirty plants of each genotype were measured. The error bars represent the SD. (TIF) [file pgen.1003954.s001.tif]

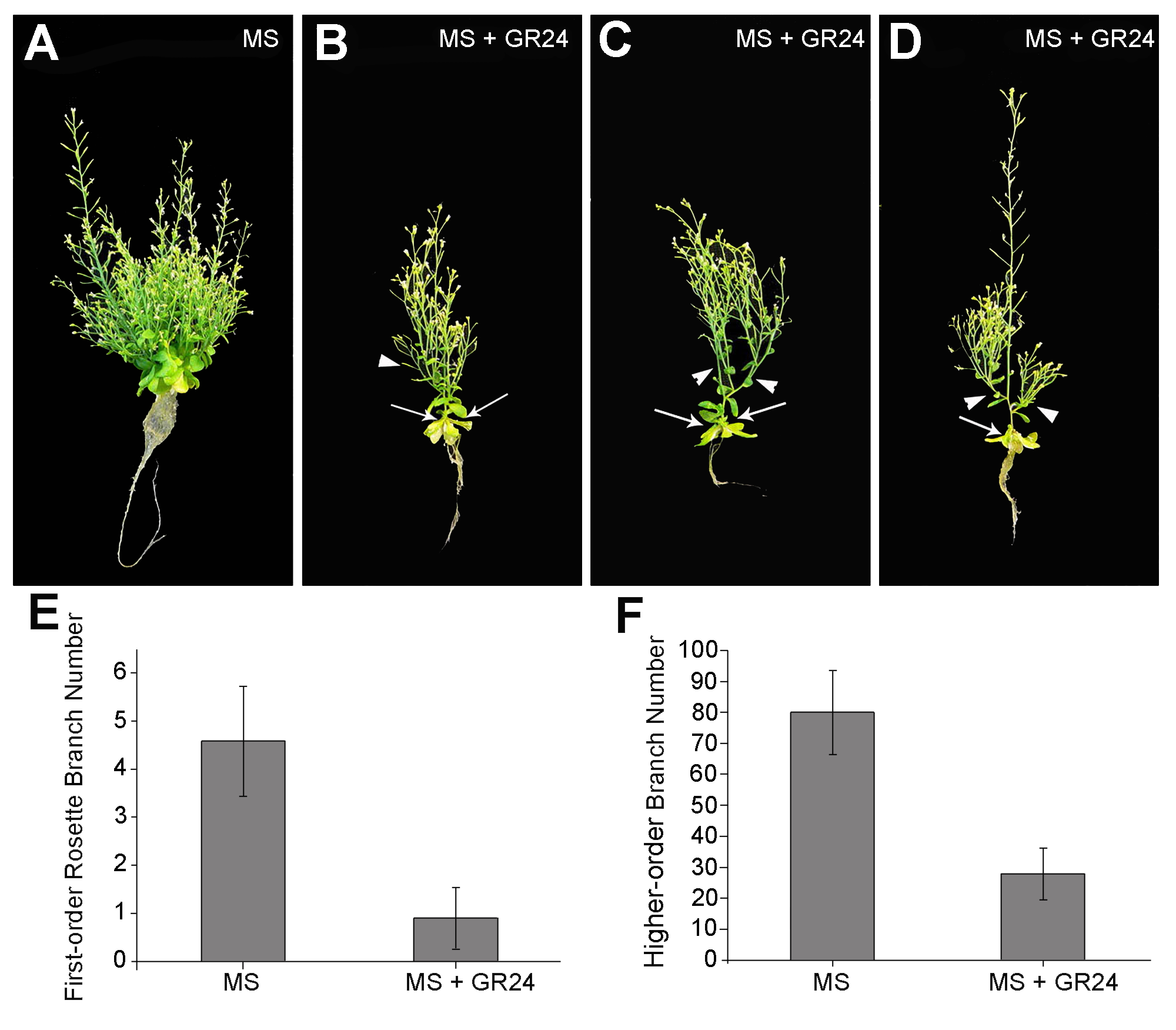

Supplement: Figure S2 — GR24 treatment of adp1-D. (A) to (D) Morphology of adp1-D grown on MS with 0.1% acetone (A) or 5 µM of GR24 (B to D) for 40 days. GR24 treatment resulted in almost completely inhibition of first-order rosette branches in adp1-D, indicated by white arrows from (B) to (D). However, GR24 had little effect on higher-order cauline branches, indicated by white arrowheads. (A) First-order rosette branch number of adp1-D grown on MS with 0.1% acetone or 5 µM of GR24. Forty plants were measured. Error bars represent SD. (B) Higher-order branch number of adp1-D grown on MS with 0.1% acetone or 5 µM of GR24. Forty plants were measured. Error bars represent SD. (TIF) [file pgen.1003954.s002.tif]

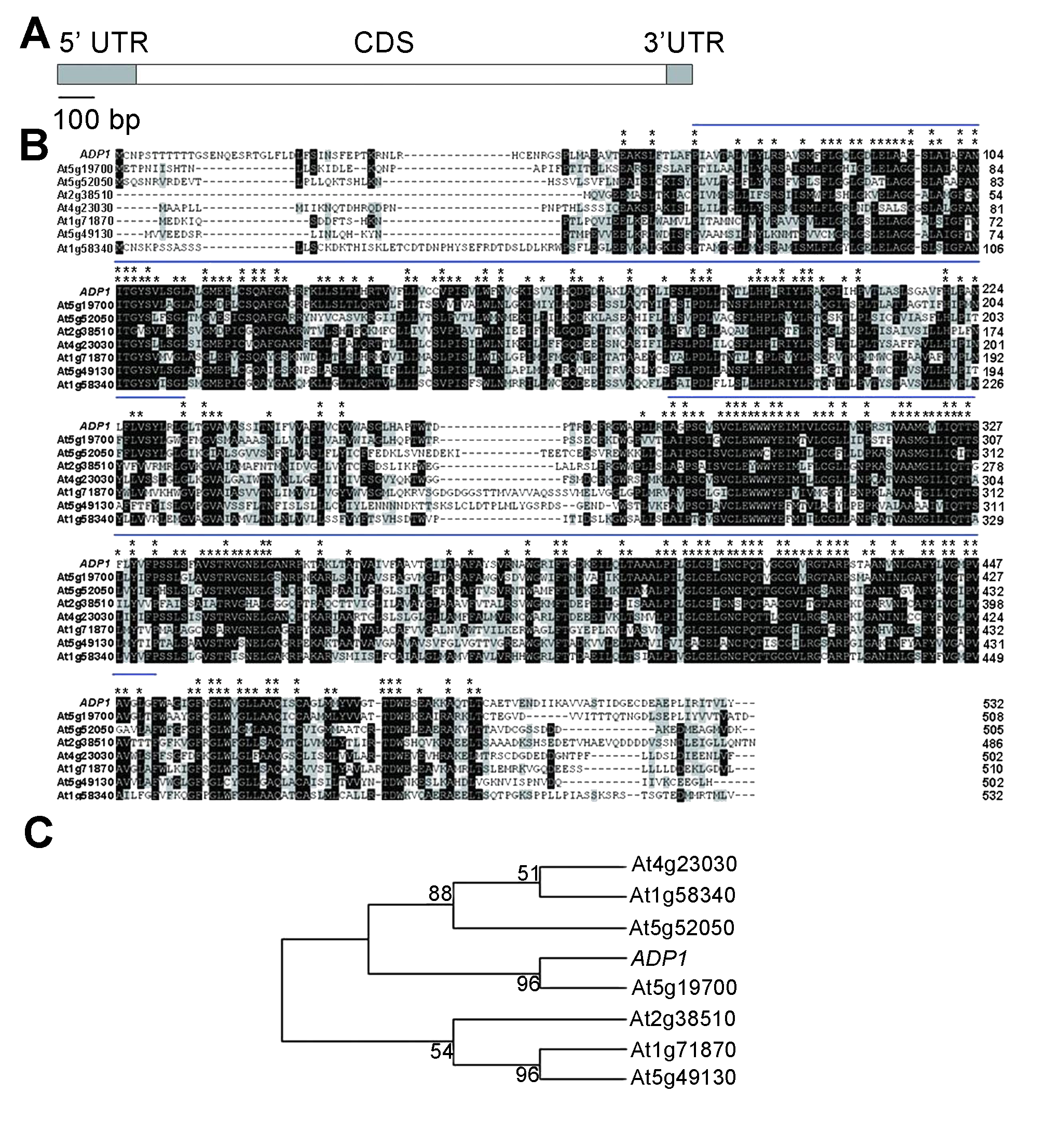

Supplement: Figure S3 — ADP1 belongs to the MATE transporter family. (A) Schematic diagram of the ADP1 cDNA structure. Grey bars represent the untranslated regions (UTR), the white bar represents the coding sequence (CDS). (B) Sequence alignment of eight genes belonging to the same clade as ADP1. Double asterisks indicate high identity, and a single asterisk indicates moderate similarity. The blue line indicates the sequence of the MATE domain. (C) Phylogenic relationships among eight genes from the same clade as ADP1. (TIF) [file pgen.1003954.s003.tif]

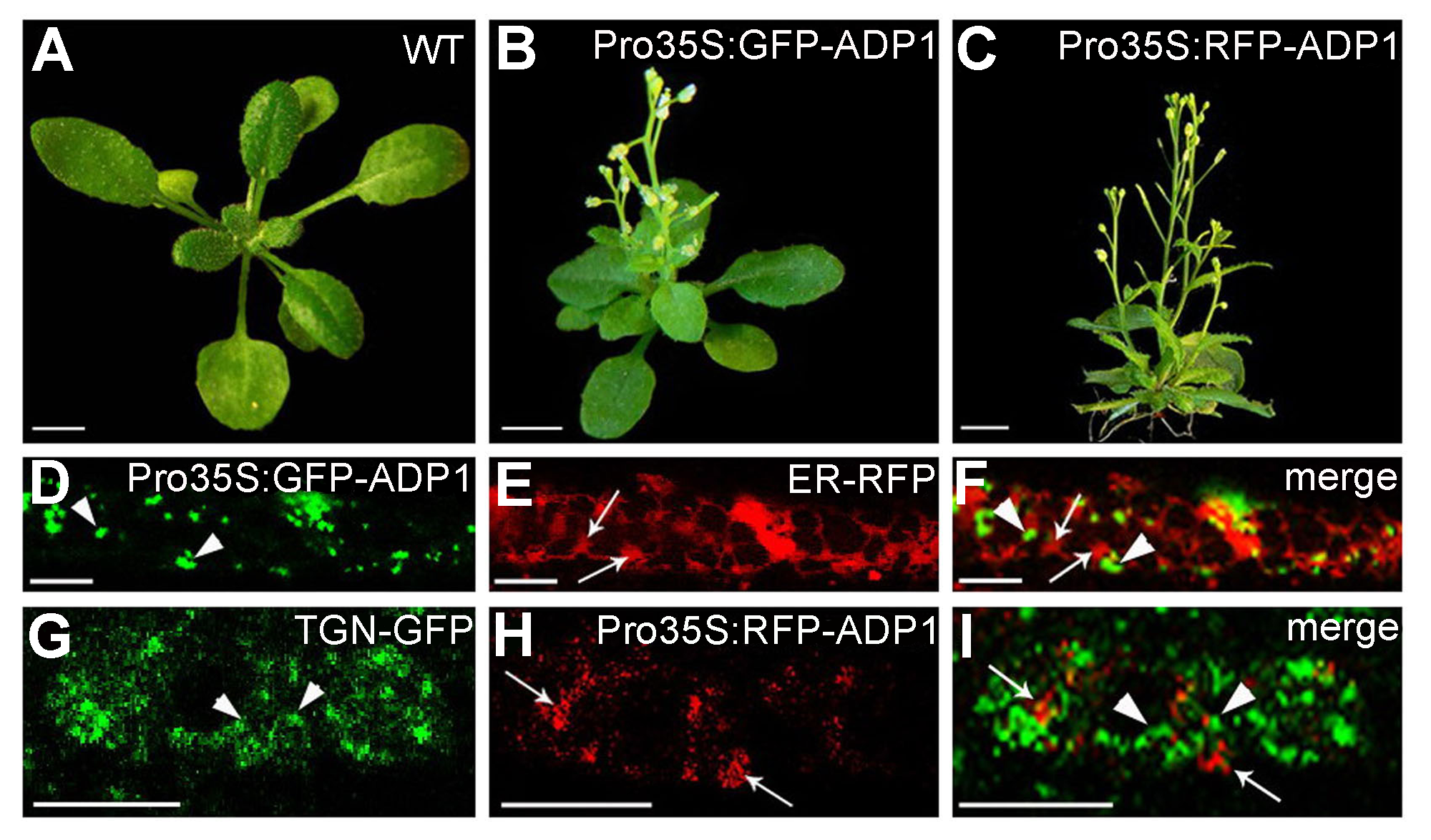

Supplement: Figure S4 — Co-localization of ADP1 with marker lines. (A) to (C) Morphology of 30 days' wild type plants (A), transgenic plants of Pro35S:GFP-ADP1 (B) and Pro35S:RFP-ADP1(C). (D) to (F) The fluorescent signal of GFP-ADP1 did not co-localize with ER-RFP, as indicated by arrowheads (GFP-ADP1) and arrows (ER-RFP). Bar = 20 µm. (G) to (I) The fluorescent signal of RFP-ADP1 did not co-localize with TGN-GFP, as indicated by arrowheads (TGN-GFP) and arrows (RFP-ADP1). Bar = 20 µm. (TIF) [file pgen.1003954.s004.tif]

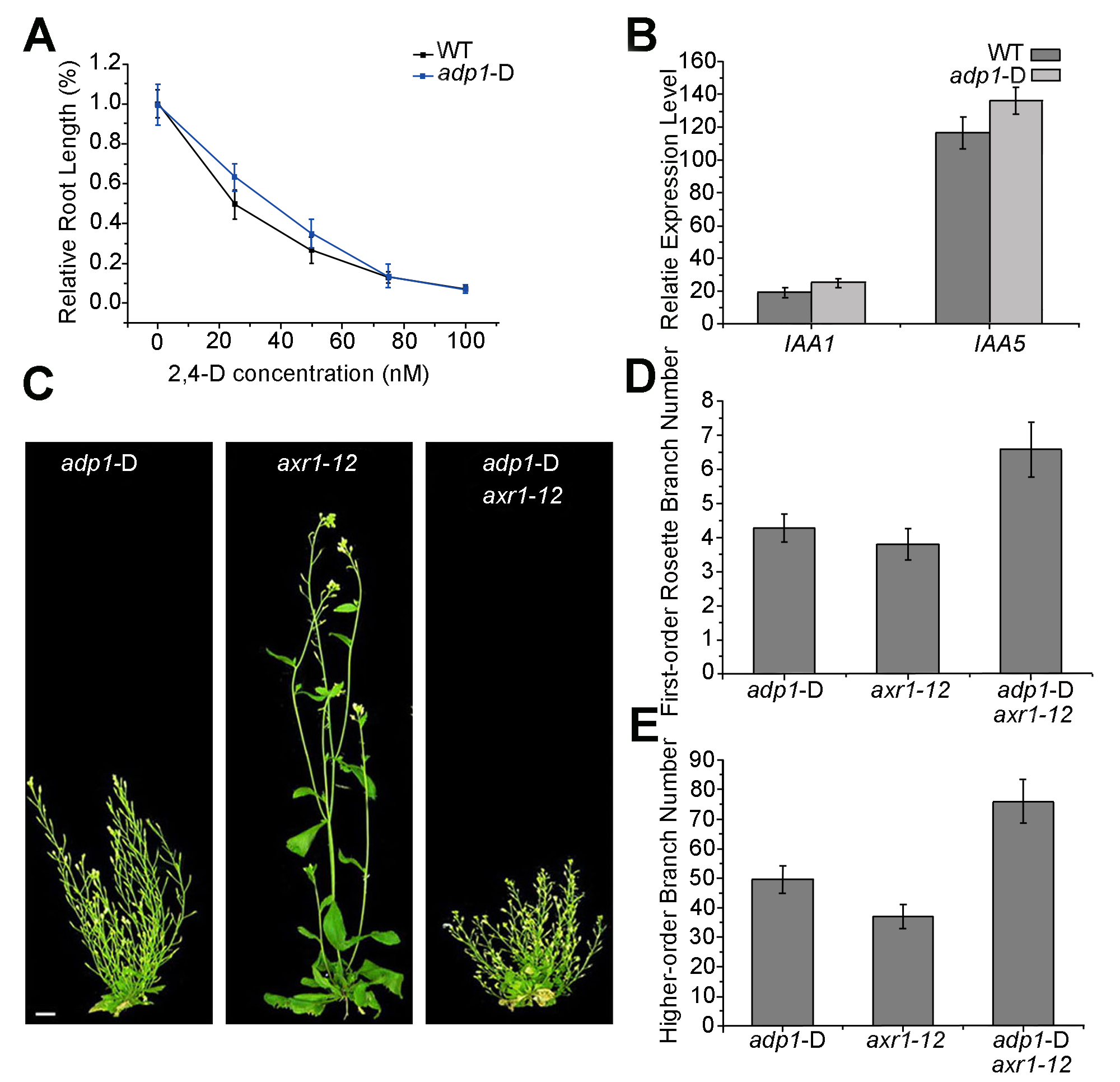

Supplement: Figure S5 — Auxin signal transduction pathway in adp1-D. (A) Primary root length in wild-type and adp1-D seedlings after growth on medium containing different concentrations of 2,4-D for 6 days. At least 30 seedlings were measured for each genotype. The error bars represent the SD. (B) IAA1 and IAA5 expression in wild-type and adp1-D seedlings after 1 h treatment with 20 µM 2,4-D. (C) Phenotypes of six-week-old adp1-D, axr1-12, and adp1-D axr1-12 mutants. Bar = 1 cm. (D) First-order rosette branch number in the wild type, adp1-D, axr1-12, and adp1-D axr1-12. (E) Higher-order branch number in the wild type, adp1-D, axr1-12, and adp1-D axr1-12. For (D) and (E), at least 30 seedlings were measured for each genotype. The error bars represent the SD. (TIF) [file pgen.1003954.s005.tif]

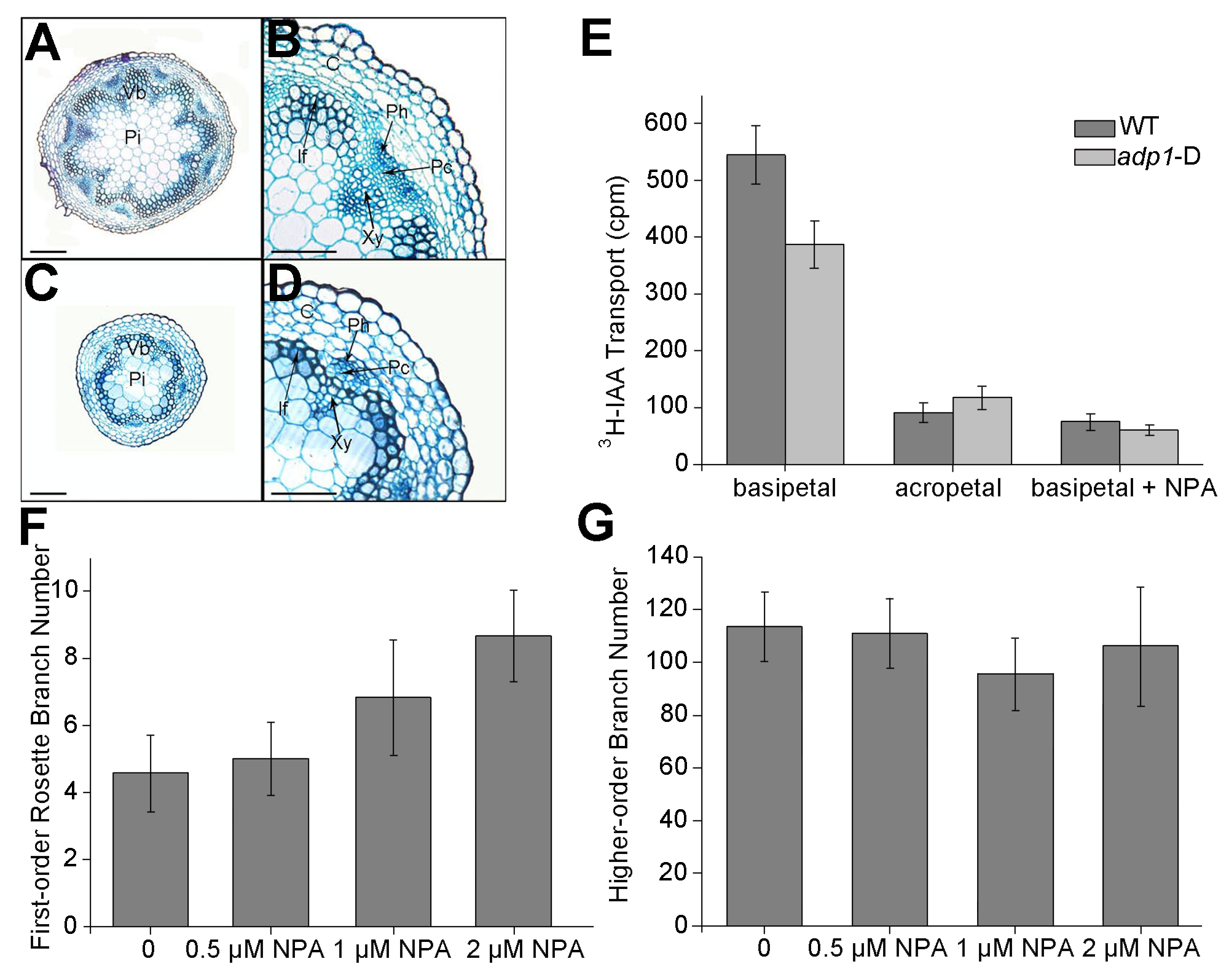

Supplement: Figure S6 — Auxin flux in the main stem did not change in adp1-D. (A), (B), (C), and (D) Transverse sections of vascular tissue in the basal portion of the inflorescence stem of A) and B) wild-type and C) and D) adp1-D plants stained with toluidine blue. C, cortex; If, inter-fascicular fiber; Pc, (pro) cambium; Pi, pith; Ph, phloem; Vb, vascular bundle; Xy, xylem. Bars = 100 µm. (B) and (D) Higher-magnification images of the vascular tissue in B) the wild type and D) adp1-D. (E) Polar auxin transport in the inflorescence stem of wild-type and adp1-D homozygous seedlings. Fifteen seedlings of each genotype were assayed. Values shown are means ± SD. (F) and (G) Measurement of first-order rosette branch number (F) and higher-order branch number (G) of adp1-D plants after cultivation for six weeks on MS medium containing 0.1% DMSO, 0.5 µM, 1 µM, or 2 µM NPA. Twenty plants were measured in each treatment. (TIF) [file pgen.1003954.s006.tif]

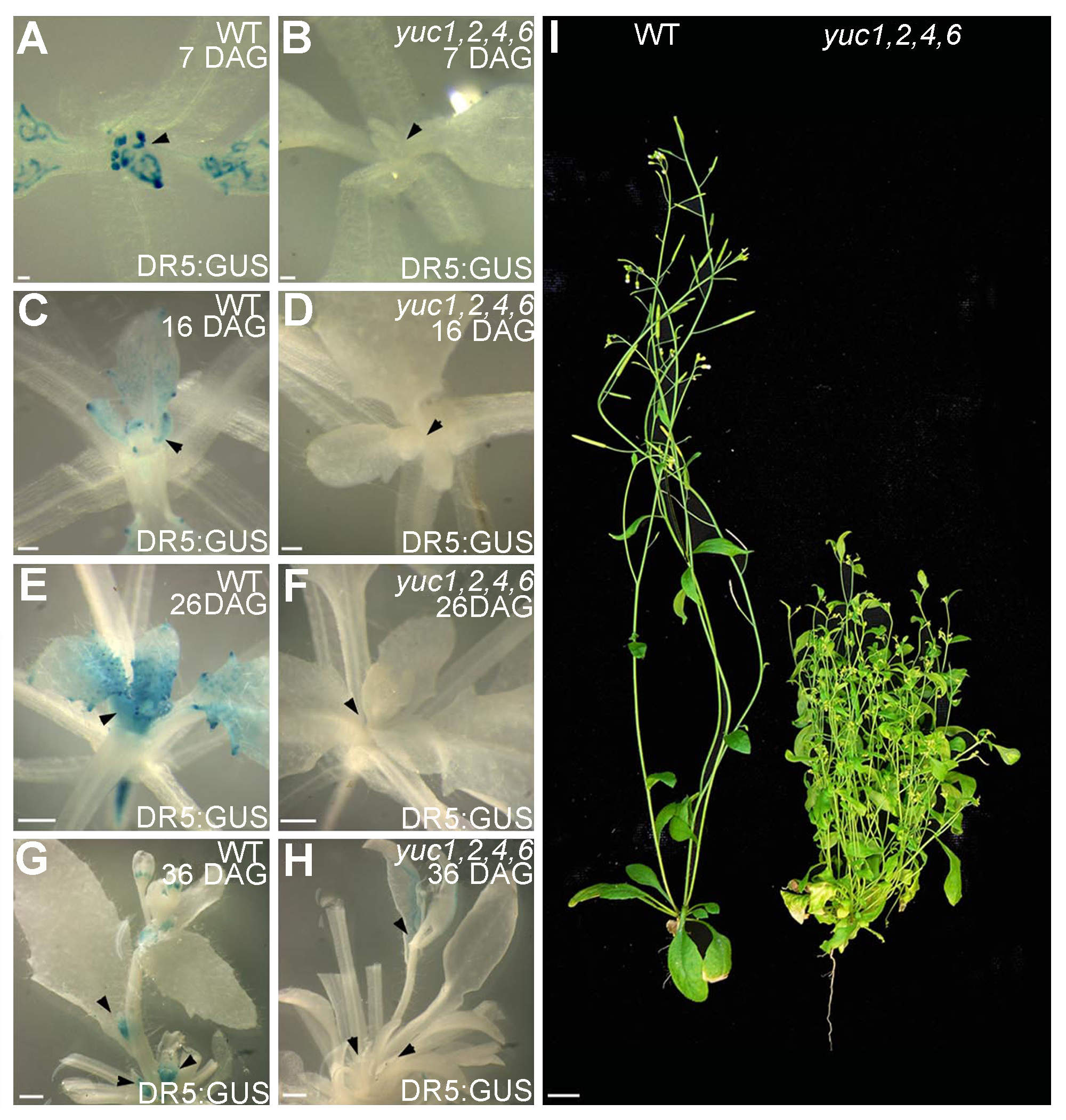

Supplement: Figure S7 — Reduction in DR5:GUS signal in yuc1,2,4,6 quadruple mutants. (A) to (H) DR5:GUS signal at different developmental stages in the wild type (A, C, E, G) and yuc1,2,4,6 mutants (B, D, F, H). The GUS signal (indicated by black arrowheads) was almost undetectable in meristematic regions in the quadruple mutants compared with that in the wild type. For (A) to (F), bar = 0.1 mm; (G) and (H), bar = 0.4 mm. (I) Phenotypes of 2-month-old wild type and yuc1,2,4,6 plants. Bar = 1 cm. (TIF) [file pgen.1003954.s007.tif]

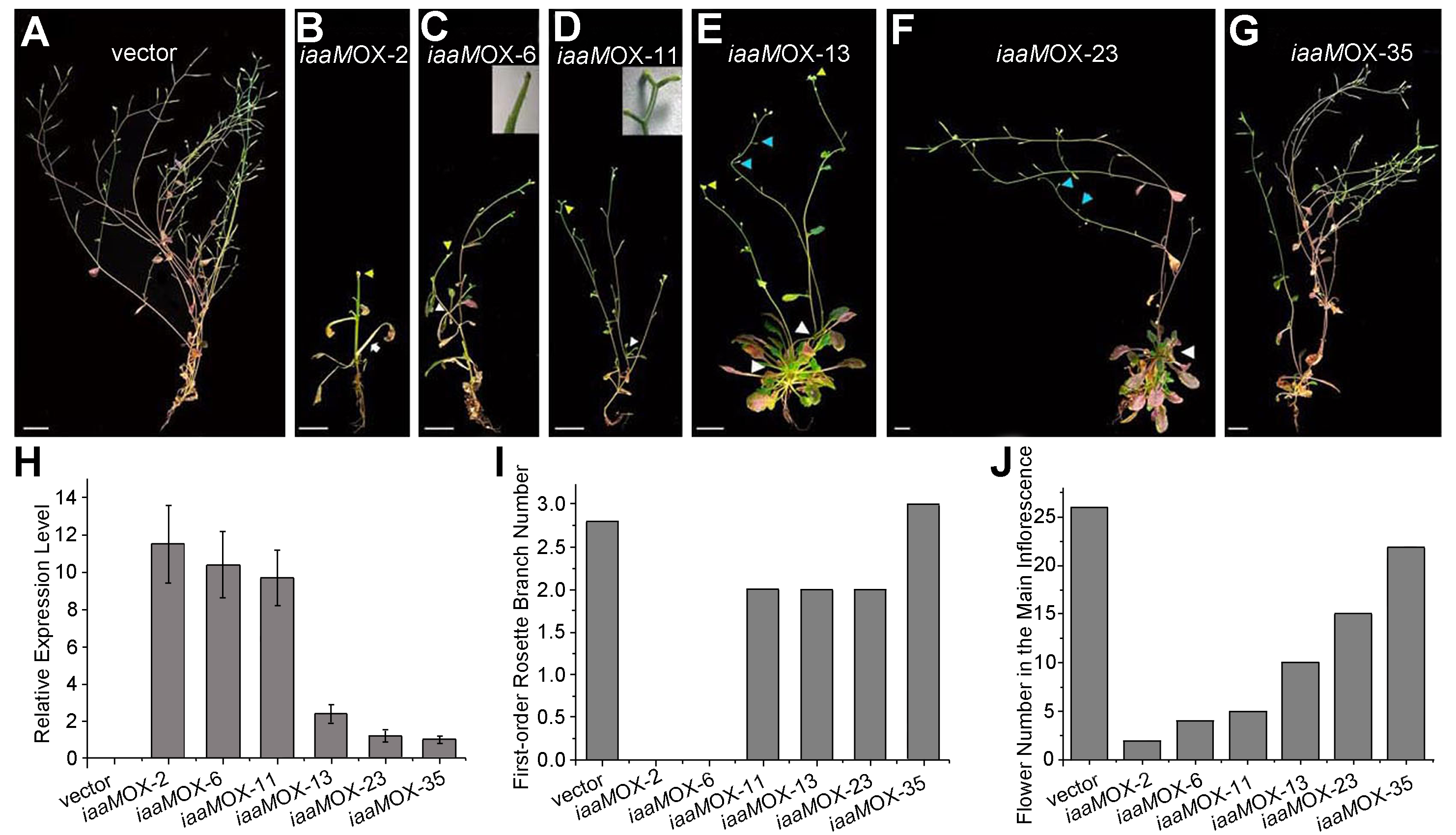

Supplement: Figure S8 — Correlation of auxin level with lateral organ number. (A) to (G) Morphology of transgenic plants carrying the control vector (A) or ProADP1:iaaM. The epinastic leaf is indicated with a white arrow; aerial rosette leaves are indicated by white arrowheads; terminated shoot tips are indicated by yellow arrowheads; and sterile siliques are indicated by blue arrowheads. Bar = 1 cm. (H) Expression quantity of iaaM in the transgenic lines shown in (A) to (G) analyzed by real-time-qPCR. (I) First-order rosette branch number of transgenic lines shown in (A) to (G). (J) Flower number on the main of transgenic lines shown in (A) to (G). (TIF) [file pgen.1003954.s008.tif]

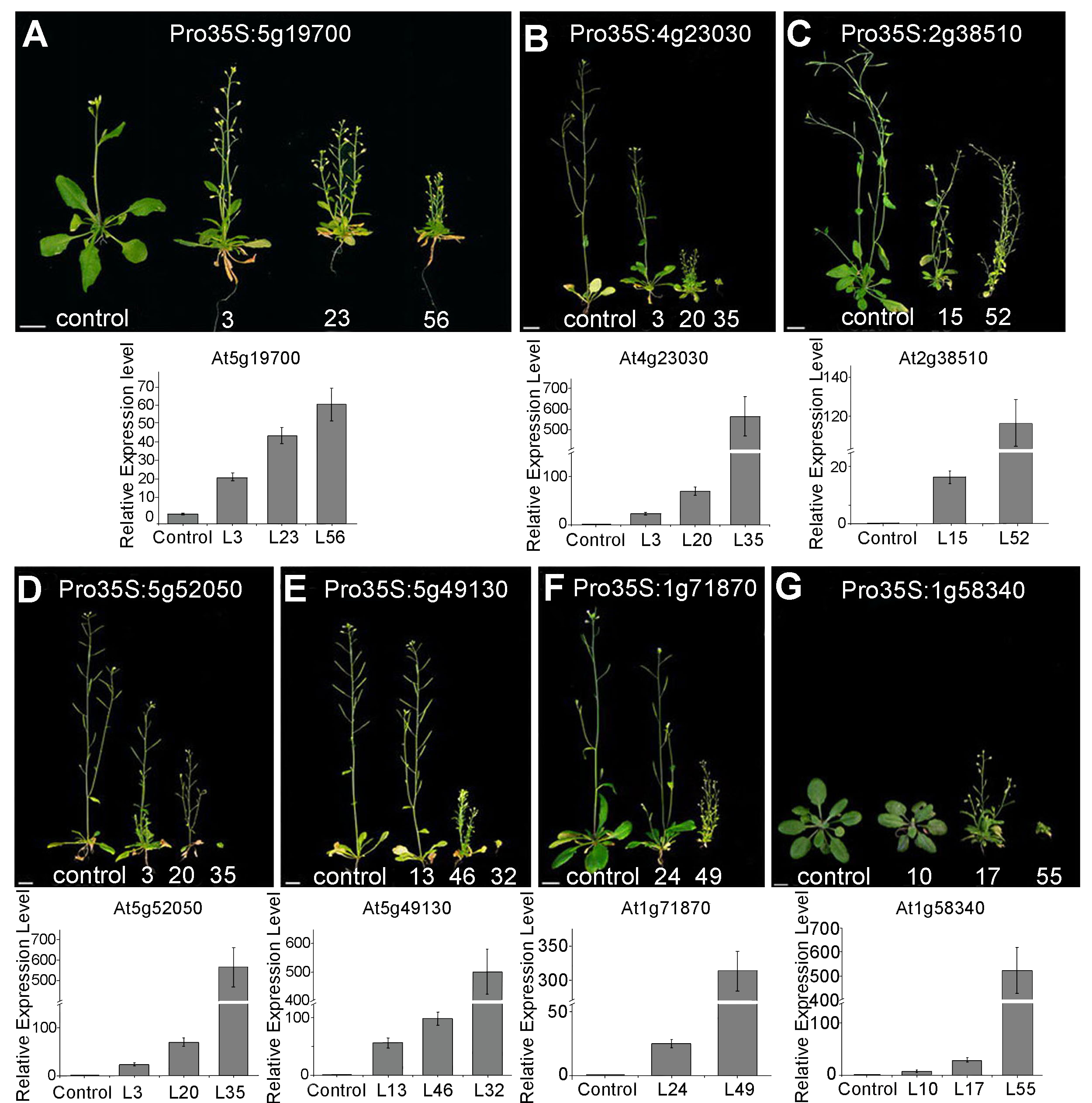

Supplement: Figure S9 — Recapitulation of adp1-D phenotypes by over-expression of genes in the same clade. (A) to (G) Phenotypes of transgenic plants over-expressing genes belonging to the same clade as ADP1 under the CaMV 35S promoter and the expression quantity in the corresponding lines (see legend below). All transgenic plants recapitulated the bushy and accelerated growth rate phenotypes of adp1-D to different extents. The expression level was in accordance with the severity of the phenotype. Phenotypes and expression level of transgenic plants over-expressing A) Pro35S:5g19700, B) Pro35S:4g23030, C) Pro35S:2g38510, D) Pro35S:5g52050, E) Pro35S:5g49130, F) Pro35S:1g71870 and G) Pro35S:1g58340. Bar = 1 cm. (TIF) [file pgen.1003954.s009.tif]

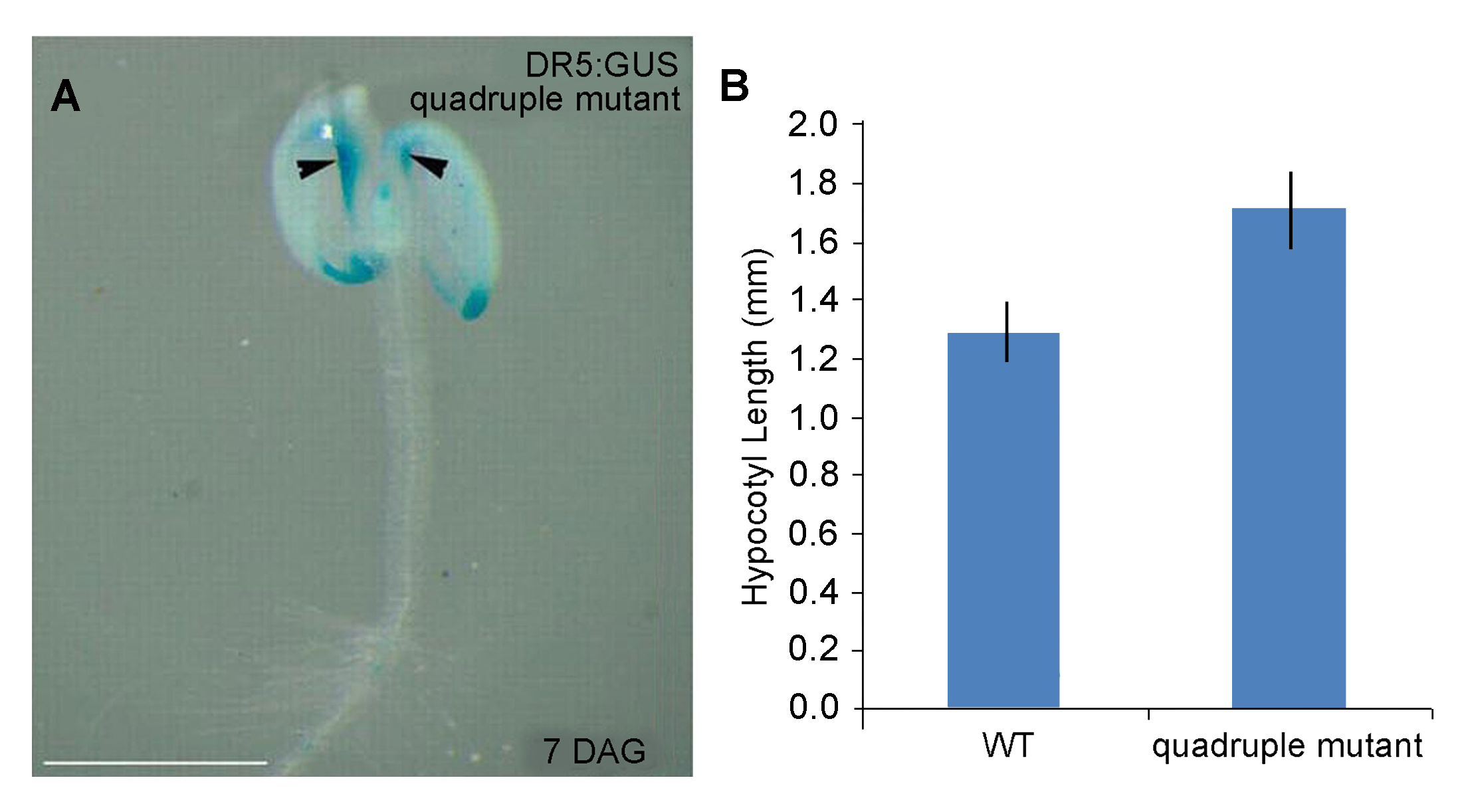

Supplement: Figure S10 — The phenotypes of quadruple mutant. The quadruple mutant exhibited epinastic cotyledon (A) and increased hypocotyl length (B). Black arrows indicate epinastic cotyledons. The error bars represent the SD. Bar = 1 mm. (TIF) [file pgen.1003954.s010.tif]
