# Supplementary material for: ADP1 Affects Plant Architecture by Regulating Local Auxin Biosynthesis
Source: PLoS Genet. 2014 Jan 2;10(1):e1003954. doi: 10.1371/journal.pgen.1003954 (PMC3879159; doi:10.1371/journal.pgen.1003954)
Supplement: Table S1 — IAA precursors and IAA levels (pg (ngΔ)/mg FW). (DOC) [file pgen.1003954.s011.doc]

| **Table S1.** IAA precursors and IAA levels (pg (ng^Δ^)/mg FW) | | | | | | |
| --- | --- | --- | --- | --- | --- | --- |
| materials | IAN^Δ^ | IAM | IPyA | IAAld | IAA |  |
| WT seedlings | 43±4 | 3.3±1.0 | 125±9 | 94±15 | 50.9±2.7 |  |
| *adp1-D* seedlings | 41±1 | 4.0±0.2 | 269±184 | 121±52** | 25.1±19.5 |  |
| quadruple seedlings | 62±12 | 5.4±1.8 | 313±95 | 90±18 | 40.4±13.3 |  |
| WT axillary buds | 122±9 | 10.1±0.9 | 645±183 | 244±64 | 12.9±2.8 |  |
| *adp1-D* axillary buds | 43±15** | 9.0±1.2 | 387±259 | 55±17 | 6.1±1.2* |  |
| quadruple axillary buds | 149±25 | 13.4±1.6* | 803±130 | 195±68 | 13.3±0.5 |  |

The data are shown as mean values ± one SD and student’s *t*-test was performed (*P<0.05 and **P<0.01).
